# CsdA‐LaeB Regulatory Hub Contributes to *Aspergillus fumigatus* Virulence via Fumiquinazoline C Biosynthesis

**DOI:** 10.1002/advs.202519021

**Published:** 2026-01-07

**Authors:** Zili Song, Hongjiao Zhang, Leixin Ye, Yuxin Lei, Linqi Wang, Xiao Liu, Nayanna M. Mercado Soto, Nancy P. Keller, Berl R. Oakley, Can Zhao, Michael Bromley, Hongwei Liu, Lei Cai, Koon Ho Wong, Wen‐Bing Yin

**Affiliations:** ^1^ State Key Laboratory of Microbial Diversity and Innovative Utilization, Institute of Microbiology Chinese Academy of Sciences Beijing China; ^2^ Medical School University of Chinese Academy of Sciences Beijing China; ^3^ Department of Medical Microbiology and Immunology University of Wisconsin‐Madison Madison Wisconsin USA; ^4^ Department of Molecular Biosciences University of Kansas Lawrence Kansas USA; ^5^ Manchester Fungal Infection Group Division of Infection, Immunity and Respiratory Medicine University of Manchester Manchester UK; ^6^ Department of Life Sciences Manchester Metropolitan University Manchester UK; ^7^ MoE Frontiers Science Center for Precision Oncology University of Macau Macau SAR China

**Keywords:** clinical isolates, fungal virulence, gene regulation, RNA‐binding protein, secondary metabolite

## Abstract

Fungal secondary metabolism plays a critical role in pathogen–host interactions, yet the regulatory networks linking metabolic reprogramming to virulence remain poorly understood. This study identifies a conserved regulatory hub in the human pathogen *Aspergillus fumigatus*, where the RNA‐binding protein (RBP) CsdA interacts with the global regulator LaeB in the nucleus to regulate biosynthesis of the secondary metabolite fumiquinazoline C (FqC). Disruption of the CsdA‐LaeB interaction hyperactivates FqC production, enhancing fungal colonization and lethality in murine invasive aspergillosis models. Integrative metabolomic and transcriptomic analyses reveal that CsdA and LaeB function as co‐regulators of a broader secondary metabolic gene cluster network, with FqC emerging as an effector that mediates virulence in vivo. Genetic validation confirms that FqC is strictly required for the increased virulence phenotype of CsdA‐ or LaeB‐deficient strains, while analyses of clinical isolates demonstrate a striking inverse correlation: reduced CsdA and LaeB expression coincides with elevated FqC production, showing consistency with the infection outcomes of the deletion mutants. This work identifies the RBP‐based interaction that regulates fungal metabolic virulence, shedding new light on the post‐transcriptional regulatory logic linking secondary metabolism to pathogenicity and offering alternative strategies for diagnostic development and therapeutic intervention in invasive fungal diseases.

## Graphical abstract

This study identifies a conserved CsdA‐LaeB hub in *Aspergillus fumigatus* that regulates secondary metabolite fumiquinazoline C (FqC). Disrupting this hub enhances FqC production and virulence, shedding new light on the post‐transcriptional regulatory logic linking secondary metabolism to pathogenicity and offering alternative strategies for diagnostic development and therapeutic intervention in invasive fungal diseases.

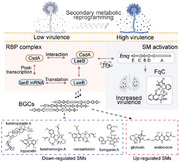

## 1 Introduction

Invasive aspergillosis, a life‐threatening fungal infection primarily caused by the opportunistic pathogen *Aspergillus fumigatus*, represents a significant global health burden and remains a leading cause of mortality [1, 2, 3, 4, 5]. Annually, over 2 million individuals develop invasive aspergillosis, primarily in the context of chronic obstructive pulmonary disease (COPD), intensive care, lung cancer, or hematological malignancies, resulting in an estimated crude annual mortality of 1.8 million [6]. *Aspergillus fumigatus*, a formidable human pathogen, predominantly infects immunocompromised individuals, such as cancer patients, transplant recipients, and those grappling with COPD [7, 8]. Notably, during host‐pathogen interactions, bioactive secondary metabolites (SMs) produced by the invading *A. fumigatus* play a crucial role in modulating immune responses [9]. For example, DHN‐melanin localizes to the conidial surface, where it protects conidia by scavenging reactive oxygen species (ROS), reducing phagolysosomal acidification in alveolar macrophages, and inhibiting epithelial cell apoptosis [10]. Concurrently, DHN‐melanin is specifically recognized by the C‐type lectin receptor MelLec and plays a critical role in protective antifungal immunity in mice and humans [11]. Gliotoxin inhibits the activity of NADPH oxidase, diminishes the cytotoxicity of T lymphocytes, and additionally suppresses phagocytic activity in macrophages [12]. Additionally, siderophores produced by *A. fumigatus*, including fusarinine C and triacetylfusarinine C, sequester iron ions in competition with the host to meet the fungus's micronutrient needs [13]. However, the functions of multiple SMs and the regulatory networks governing their biosynthesis during infection remain poorly understood.

Fungal SMs are encoded by biosynthetic gene clusters (BGCs). In *A. fumigatus*, 20 BGCs have been linked to specific SMs [14]. SM production is regulated by complex hierarchical networks, that include pathway‐specific, epigenetic, and global regulation [12, 15, 16]. Several pathway‐specific transcription factors (e.g., GliZ for gliotoxin), epigenetic modifiers (e.g., histone deacetylase RpdA) and global regulators (e.g., LaeA, control approximately 50% BGCs) have been characterized [17, 18, 19, 20]. RNA‐binding proteins (RBPs) emerge as a novel class of regulators influencing eukaryotic post‐transcriptional modifications, are well studied in human disease [21, 22] and plant development [23, 24], yet their roles in fungal metabolism and virulence are veiled in an enigma. Furthermore, although global regulators such as LaeB are known to coordinate BGC activation [25, 26], their interplay with RBPs and relevance to fungal virulence remain uncharted.

Here, we bridge this gap by integrating pan‐metabolomics and functional genetics to unravel a novel regulatory hub that links fungal metabolic virulence. Through pan‐metabolome analysis, we identified distinct metabolic profiles between clinical and environmental *A. fumigatus* isolates, with fumiquinazoline C (FqC) emerging as a main contributor to virulence within the CsdA/LaeB regulatory network. Transcriptomic and metabolomic analyses revealed an unknown interaction between CsdA and LaeB in the nucleus, confirmed by in vitro pull‐down and in vivo bimolecular fluorescence complementation assays. Strikingly, disruption of this interaction hyperactivated FqC production, increasing fungal colonization and virulence, revealing a delicate balance between metabolic regulation and host adaptation. Genetic validation underscores FqC as the primary metabolite contributing to the increased virulence of CsdA/LaeB‐deficient strains, while clinical isolates mirror this regulatory hub—displaying blunted CsdA/LaeB expression that parallels heightened FqC production, hinting at a conserved virulence‐regulating strategy in *A. fumigatus*. This study innovatively uncovers RBP as orchestrator of fungal secondary metabolism to adapt to the host pulmonary microenvironment, offering a fresh perspective that bridges the gap between secondary metabolism regulatory networks and fungal pathogenic strategies.

## 2 Results

### 2.1 Metabolic Divergence Between Clinical and Environmental *A. fumigatus* Isolates

**FIGURE 1:**
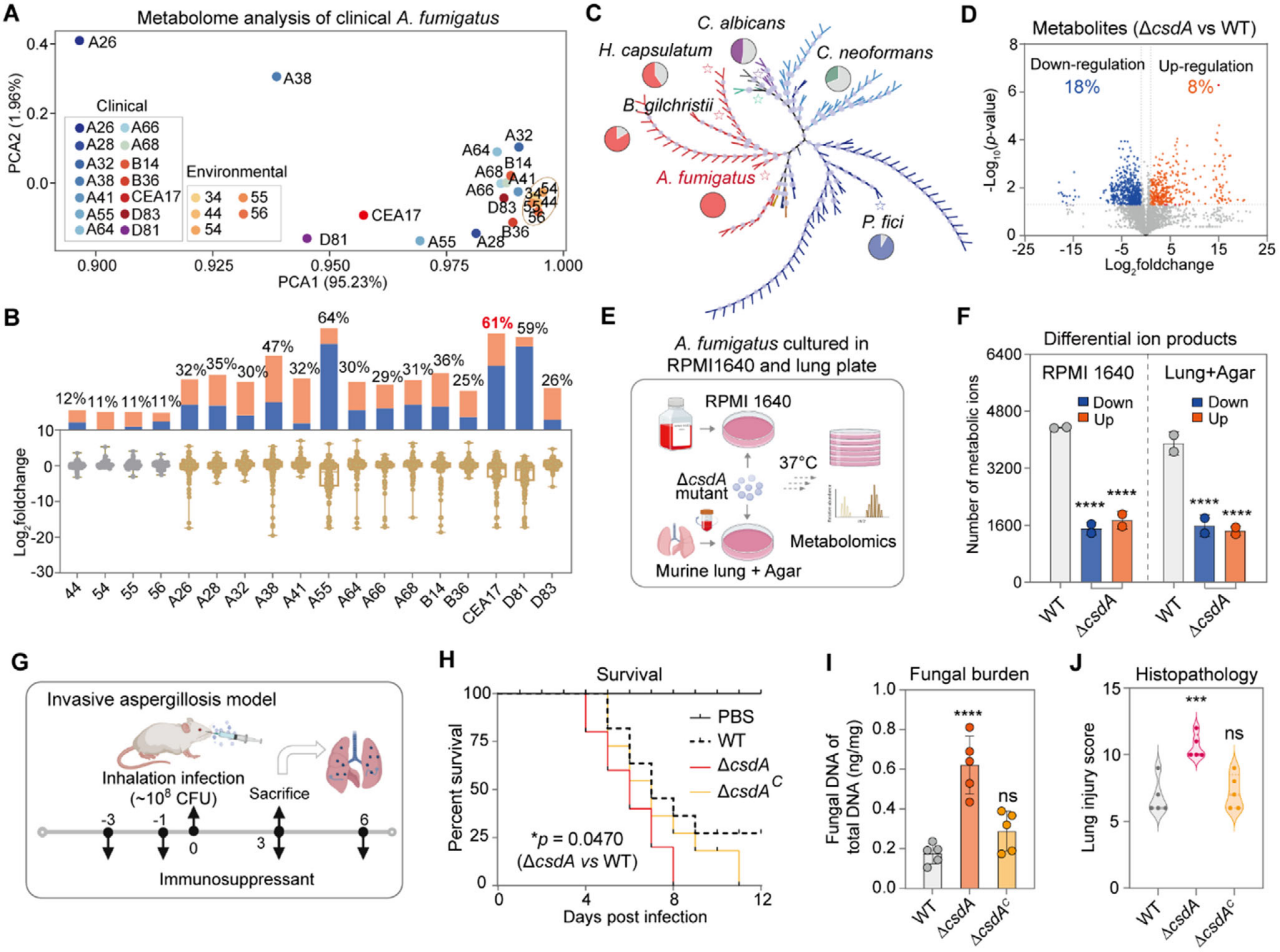
Metabolic profiling analysis established the connection between RNA‐binding protein CsdA‐mediated metabolism and virulence in *Aspergillus fumigatus*. A) Comparative metabolomics analysis of clinical and environmental *A. fumigatus*. Circles represent the metabolic profiles of environmental strains that cluster together, whereas clinical strains exhibit metabolic diversity. The pathogenic information of the clinical isolates used is listed in Table S6. B) Differential metabolite production in all *A. fumigatus* isolates compared to environmental strain 34. Percentages represent the total proportion of differential metabolites; orange indicates metabolites with increased abundance, and blue indicates those with decreased abundance. C) Phylogenetic tree of RNA binding protein CsdA in pathogenic fungi. The pie chart shows the sequence identity of CsdA homologous proteins across several representative pathogenic fungi. All homologous proteins are listed in Table S2. D) Volcano plots showing the differentially regulated metabolic ion products in the Δ*csdA* mutant versus the control. |Log_2_foldchange|>1 and ‐Log_10_(*p*‐value)>1.3 indicate a significant difference. E) Schematic of metabolomics analysis of Δ*csdA* mutant cultured in RPMI1640 and lung plate medium. F) Comparative metabolic analysis of the Δ*csdA* mutant and the control strains in RPMI 1640 and lung plate medium. Blue represents metabolites with decreased abundance in the mutant strains, while orange represents those with increased abundance. G) Workflow for evaluating Δ*csdA* mutant virulence in invasive aspergillosis model. Figure 1, G) were created in BioRender. Song, Z. (2025) https://BioRender.com/dvhyfw4. H) Survival curves of Balb/c mice intranasally infected With *csdA* deletion and complementation mutants compared with control strains (*n* = 10, log‐rank test). All three independent replicate experiments produced consistent results, and only one experiment result is presented in the figure. I) Fungal burden in the lungs of *A. fumigatus* and its mutant after 3 d of infection (*n* = 5). J) Histopathological assessment of lung injury on day 3 postinfection with *A. fumigatus* and its mutant (*n* = 5). Quantification of lung tissue injury was performed according to the Smith scoring criteria. All error bars are expressed as mean ± SD. Statistical analysis was performed by using one‐way ANOVA (“ns”: not significant. Significant at *p < 0.05, ***p < 0.001, ****p < 0.0001).

To explore the link between *A. fumigatus* metabolism and virulence, we performed a non‐targeted pan‐metabolomics analysis of 19 *A. fumigatus* isolates, including 5 environmental isolates (from soil and water environments), 13 clinical isolates (from 10 patients with invasive pulmonary disease and 3 with chronic pulmonary disease), and model strain CEA17 (as also clinical isolate) (Table S1). Total ion peaks give a good indication of the relative number of metabolites produced. Principal components analysis (PCA) revealed significant metabolic differences between clinical and environmental isolates (Figure 1A). To quantify the metabolic differences between the two groups, we compared the abundance of each detected ion across isolates using the environmental isolate 34 as a reference. Quantitative profiling revealed 11%‐12% of detected metabolic ion products exhibited abundance variations in all other environmental isolates. Strikingly, clinical isolates demonstrated substantially greater divergence, with 25%‐64% of metabolic ion products differing from environmental isolate 34, including a 61% variation in the model strain CEA17 (Figure 1B). These findings indicate greater metabolic diversity among clinical *A. fumigatus*.

### 2.2 RNA‐Binding Protein CsdA Governs Global Metabolism and Virulence

Fungal metabolism is usually controlled by a series of key regulatory proteins. We recently discovered an RNA‐binding protein (RBP) CsdA in the endophytic fungus *Pestalotiopsis fici* that is critical for normal growth and metabolism [27]. RBPs play crucial roles in regulating pathogenicity in various eukaryotes, yet their mechanisms of action in fungal pathogenicity remain unclear. Analysis of fungal genome data revealed CsdA homologues existing in fungal kingdom, including in pathogenic fungi such as *Aspergillus spp*., *Histoplasma capsulatum*, *Blastomyces gilchristii*, and dermatophytes (*Arthroderma uncinatum, Trichophyton rubrum, T. mentagrophytes*), *Candida auris* and *C. albicans* (Figure 1C; Figure S1). Interestingly, the identity/coverage of CsdA homology in rare fungal pathogen *B. gilchristii* was 83.76%/100% relative to the *A. fumigatus* homolog, further supporting the presence of CsdA homologs across fungal kingdom (Figure 1C). Basidiomycete fungi (*Cryptococcus neoformans var. grubii*) exhibited lower identity/coverage (30%/10%) but retained similar RNA recognition motif (RRM) and zinc finger (ZnF) domains (Table S2).

To examine the function of CsdA, a comparative metabolome analysis between the Δ*csdA* mutant and control CEA17 strain revealed that 1321 (26% of total) ion peaks showed significant differences among the detected 5,057 products (adjusted *p* < 0.05, |log_2_foldchange|>1, Figure 1D; Table S3). To further elucidate CsdA's broad regulatory role in metabolism, we compared the metabolite profiles of Δ*csdA* mutant between RPMI1640 medium (a commonly used cell medium) and lung plate (agar supplemented mouse lung homogenate) (Figure 1E). Through a comparative metabolomics analysis, we found that the metabolic ion products regulated by CsdA in RPMI1640 medium (75%) and lung plate (78%) were significantly higher than GMM medium (26%) (Figure 1D,F). Among them, 3,252 (75% of total) ion peaks in RPMI1640 medium were regulated by CsdA, including 1,510 (35%) down‐regulated and 1,742 (40%) up‐regulated (Figure 1F). Similarly, 3,042 (78% of total) ion peaks in lung plate were regulated by CsdA, including 1,593 (41%) down‐regulated and 1,449 (37%) up‐regulated (Figure 1F). This indicates that CsdA triggers more extensive metabolic remodeling in both cell culture media and lung plates.

Given the established role of *A. fumigatus* toxins in virulence, we presumed that metabolites regulated by CsdA might impact the virulence of this pathogen. In a neutropenic murine model of invasive aspergillosis (Figure 1G), infection with the Δ*csdA* mutant resulted in 100% mortality by day 8, compared to 70% in the control strain. Complementation of *csdA* restored mortality to wild‐type levels (Figure 1H). Although the mortality of the Δ*csdA* mutant was similar to that of the control strain from day 1 to day 8 post‐infection, a significant difference emerged after day 8, suggesting an association with the secondary metabolites (SMs) secreted by the mutant strain at the later stage. Meanwhile, the Δ*csdA* mutant also had a higher fungal burden in infected murine lungs, concomitant with the higher mortality (Figure 1I). Histological examination revealed severe lung tissue damage in Δ*csdA*‐infected mice, characterized by the considerable conidial germination and invasive mycelium formation. In contrast, the control strain only exhibited limited pulmonary epithelial invasion (Figure 1J; Figure S2).

### 2.3 CsdA Interacts With LaeB in the Nucleus to Exert Its Function

**FIGURE 2:**
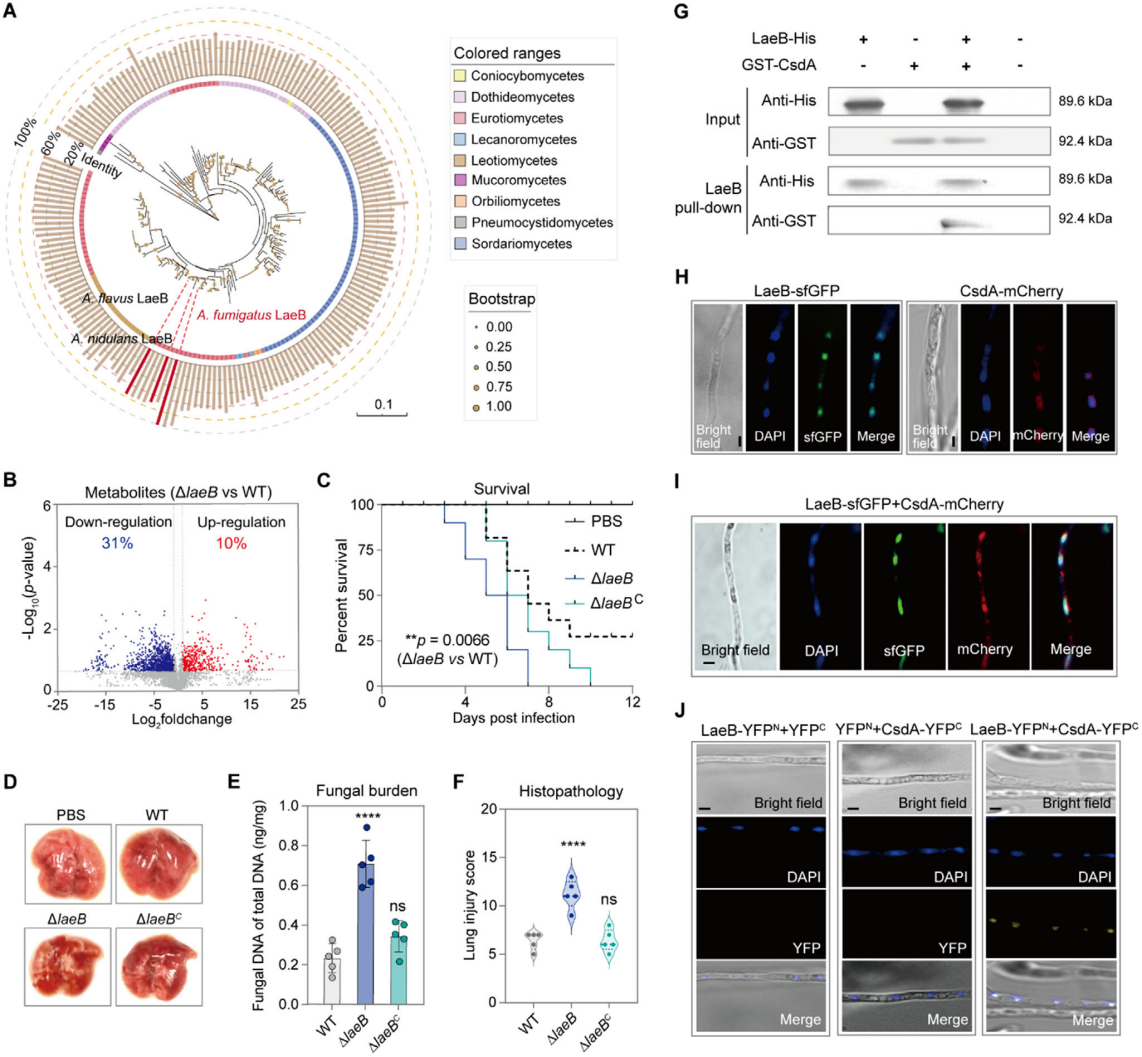
CsdA interacts with LaeB to orchestrate global metabolism and virulence. A) Phylogenetic analysis of LaeB in filamentous fungi. Red: LaeB protein (first reported in *A. fumigatus*). Strains not shown in the figure are listed in Table S2. B) Volcano plots showing the differentially regulated metabolic ion products in the Δ*laeB* mutant versus the control. |Log_2_foldchange|>1 and ‐Log_10_(*p*‐value) > 1.3 indicate a significant difference. C) Survival curves of Balb/c mice intranasally infected with *laeB* deletion and complementation mutants compared with control strains (*n* = 10, log‐rank test). Late infection stage: Mutant vs control showed distinct differences. All three independent replicate experiments produced consistent results, and only one experiment result is presented in the figure. D) Macroscopic pathology of the lung collected on day 3 post‐infected by *A. fumigatus* and its mutant. E) Fungal burden in the lungs of *A. fumigatus* and its mutant after 3 d of infection (*n* = 5). F) Histopathological assessment of lung injury on day 3 post infection with *A. fumigatus* and its mutant (*n* = 5). Quantification of lung tissue injury was performed according to the Smith scoring criteria. G) The interaction between CsdA and LaeB was studied by in vitro pull‐down assay. Localization (H) and co‐localization (I) of LaeB and CsdA in *A. fumigatus*. LaeB‐sfGFP and CsdA‐mCherry localize in the nucleus and superimpose with DAPI signals. Nuclei in hyphae were stained with 4’, 6‐diamidino‐2‐phenylindole (DAPI). Scale bars, 5 µm. J) The interaction between CsdA and LaeB was studied by in vivo bimolecular fluorescence complementation (BiFC). The strains harboring a single construct (LaeB‐YFP^N^ or CsdA‐YFP^C^) were used as negative controls. Scale bars, 5 µm. All error bars are expressed as mean ± SD. Statistical analysis was performed by using One‐way ANOVA (“ns”: Not significant. Significant at **p < 0.01, ****p < 0.0001).

Previously, we demonstrated that CsdA interacts with RsdA to regulate secondary metabolism in *P. fici* [27]. Upon systematic investigation, RsdA's ortholog protein with 49%/80% identity/coverage was found in *A. fumigatus*. Interestingly, the protein is also identified in other *Aspergillus* species including *A. flavus* [26] and *A. nidulans* [25], known as LaeB (Figure 2A; Table S2). In these species, LaeB, a protein with undefined domains, has an established role in regulating secondary metabolism, although its function in *A. fumigatus* remains uninvestigated. Thus, a Δ*laeB* mutant was constructed in the CEA17 background to characterize its function. Growth and development analysis of the mutants revealed no significant difference in growth rate between the Δ*laeB* mutant and the control strains, whereas the Δ*csdA* mutant exhibited a significantly reduced growth rate. The colony phenotypes of *A. fumigatus* mutants were consistent with the aforementioned results, indicating that CsdA regulates hyphal growth independently of LaeB. Regarding conidiation, neither mutant showed a significant difference from the control strain, demonstrating that the CsdA‐LaeB interaction has no substantial impact on the development of *A. fumigatus* (Figure S3). Subsequently, a comparative metabolomics analysis revealed a total of 5,045 ion peaks were detected, among which 2083 (41%) displayed significant changes in the Δ*laeB* mutant (adjusted *p* < 0.05, |log_2_foldchange|>1, Table S3), consistent with observations in the Δ*csdA* mutant. Notably, 1575 (31%) were significantly down‐regulated, while 508 (10%) were significantly up‐regulated (Figure 2B). In the neutropenic murine model, we observed that the Δ*laeB* mutant—similar to the Δ*csdA* mutant—also displayed a 100% fatality rate at 7 d after infection, whereas the virulence of the *laeB* complement strain was partially attenuated compared to the deletion strain (Figure 2C). Histological analysis of the murine lungs infected with the Δ*laeB* mutant also exhibited severe pathology characterized by invasive mycelium, along with a significantly heightened fungal burden when compared to the control strain (Figure 2D–F; Figure S4). This suggests a shared role of LaeB and CsdA in mediating fungal virulence.

To probe the relationship between CsdA and LaeB, we expressed LaeB‐His and GST‐CsdA fusions in *Escherichia coli* BL21 and used them in pull‐down assay (Figure S5). GST‐CsdA (92.4 kDa) was co‐purified when LaeB‐His (89.6 kDa) was pulled down using anti‐His antibody, suggesting a direct physical interaction between LaeB and CsdA (Figure 2G). Next, we analyzed the subcellular localization of CsdA and LaeB in *A. fumigatus* (Figure 2H). Fluorescent labelling of both proteins (LaeB‐sfGFP and CsdA‐mCherry) coupled with DAPI (4’,6‐diamidino‐2‐phenylindole, a DNA binding dye) staining revealed that CsdA and LaeB co‐localize in the nucleus (Figure 2I). To confirm the interaction between the two proteins in the nucleus, we created *laeB‐YFP^N^* and *csdA‐YFP^C^* fusions in *A. fumigatus* and used them in a bimolecular fluorescence complementation (BiFC) assay. We observed a yellow fluorescent signal indicating that the two proteins interact physically and the signal co‐localized with DAPI indicating that the interaction happens in the nucleus (Figure 2J). Given that CsdA, as an RNA‐binding protein, typically functions in alternative splicing of eukaryotic introns, we quantified the splicing efficiency of *laeB* introns via qRT‐PCR. Intriguingly, compared to the control strain, deletion of *csdA* significantly enhanced the splicing of intron 3 in *laeB* pre‐mRNA, with no substantial effects on the splicing of other introns (Figure S6). These results demonstrate that CsdA exerts its function by regulating *laeB* intron splicing and subsequently interacting with the translated LaeB protein.

### 2.4 CsdA/LaeB Regulates Key Secondary Metabolites, Including FqC

**FIGURE 3:**
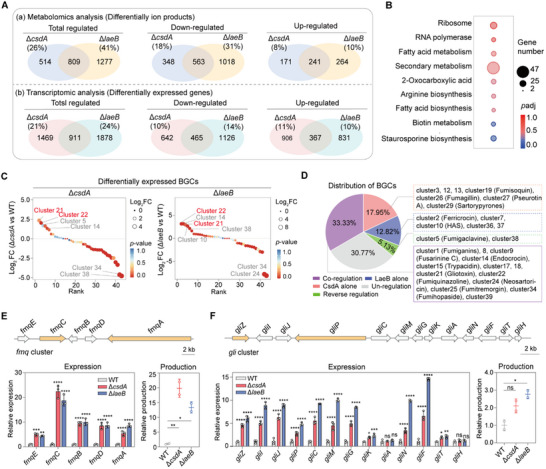
CsdA‐LaeB work together to regulate specific secondary metabolite gene clusters. A) Transcriptomic and metabolomic analysis of genes and total metabolic ion products co‐regulated by CsdA and LaeB in *A. fumigatus*. |Log_2_foldchange|>1 and ‐Log_10_(*p*‐value)>1.3 indicate a significant difference. Genes co‐regulated by the two proteins are associated with the function of the CsdA‐LaeB interaction. B) GO enrichment analysis of genes co‐regulated by CsdA and LaeB in *A. fumigatus*. C) Ranking of BGCs regulated by CsdA or LaeB based on the expression intensity of the backbone genes in *A. fumigatus*. Cluster 21: *Gli* cluster, cluster 22: *Fmq* cluster. D) The distribution of BGCs regulated by CsdA and LaeB alone or together in *A. fumigatus*. Percentages: Genes per category vs total BGCs (proportion). E,F) Representative secondary metabolites FqC (E) and gliotoxin (F) co‐regulated by CsdA and LaeB in *A. fumigatus*. The relative gene expression was detected via qRT‐PCR assay, and the product quantification was performed through metabolomic analysis. All error bars are expressed as mean ± SD. Statistical analysis was performed by using Two‐way ANOVA (“ns”: Not significant. Significant at *p < 0.05, **p < 0.01, ***p < 0.001, ****p < 0.0001).

To investigate how loss of either CsdA or LaeB increases virulence, we constructed a regulatory network co‐mediated by both proteins, involving co‐regulated metabolic ions and genes (Figure S7). Venn analysis identified 809 metabolic ions with significantly altered abundances in both Δ*csdA* and Δ*laeB* mutants (Figure 3A). Transcriptomic analysis revealed that 2380 (21%) and 2789 (24%) genes exhibited over twofold higher expression in the Δ*csdA* and Δ*laeB* mutants, respectively, compared to the control strain (Figure 3A). A total of 911 genes were significantly regulated in both mutants, predominantly related to secondary metabolism (Figure 3B). Since most SMs are encoded by biosynthetic gene clusters (BGCs), we mapped gene expression profiles of all 39 BGCs in the *A. fumigatus* genome [14, 28] (Figure S8A; Table S4). Of these, 15 (39%) backbone genes were significantly co‐regulated in both mutants, with 13 displaying the same expression trend, including 3 upregulated and 10 down‐regulated, such as *fmqC*, *encA*, *gliP*, *afumA*, *nscA*, etc. (Figure S8B–D).

In addition to gliotoxin [29, 30], *A. fumigatus* produces multiple mycotoxins [9] that modulate host immunity, such as endocrocin [31] and fumiquinazoline C (FqC) [32]. To explore how CsdA and LaeB regulate these specific mycotoxins, we systematically analyzed the BGCs controlled by the two proteins and categorized them based on the expression intensity of their backbone genes (Figure 3C). Among the most prominently affected BGCs upon *csdA* deletion, gliotoxin, fumiquinazoline, and endocrocin biosynthesis genes were up‐regulated, while fumihopaside and neosartoricin biosynthesis genes were significantly down‐regulated (Figure 3D). Interestingly, multiple transcription factors (TFs) associated with secondary metabolism or virulence, including the stress‐responsive TF SebA [32], calcium‐responsive TFs CrzA and HtfA [33, 34], pH‐responsive TF PacC [35], mitochondrial TF FhdA [36], copper homeostasis TF ClcA [37], and other TFs RsmA, ZfpA, ZfpB, and Ace2 [38, 39, 40, 41], are co‐regulated by CsdA and LaeB (Figure S9). This implies that CsdA‐LaeB interaction that mediates the transcription of multiple TFs, thereby impinging on the synthesis of diverse mycotoxins.

Specifically, fumiquinazoline production involves five genes (*fmqA‐E*) [32]. In both Δ*csdA* and Δ*laeB* mutants, *fmqA‐E* were all significantly up‐regulated by more than twofold (Figure 3E), while *sebA*, the negative TF that directly governs this gene cluster, is down‐regulated by 4.5‐ and 1.6‐ fold (Figure S9), respectively. Metabolomics analysis revealed a dramatic 19.84‐ and 13.35‐fold increase of FqC production in the Δ*csdA* or Δ*laeB* mutants, respectively, compared to the control strain (Figure 3E). To elucidate how CsdA enhances FqC production, we quantified the intron splicing efficiency of *fmqA‐E* in the Δ*csdA* mutant via qRT‐PCR. No significant differences in intron splicing of all five genes were observed compared to the control strain (Figure S6). Further analysis of the LaeB protein domain revealed no well‐defined functional domains, suggesting it does not directly bind the *fmq* gene cluster. Collectively, these results indicate that CsdA interacts with LaeB to indirectly regulate *fmq* cluster expression via SebA, ultimately modulating FqC biosynthesis. In contrast, the immunosuppressant gliotoxin production was only subtly up‐regulated by 2.11‐ and 2.77‐fold in the Δ*csdA* and Δ*laeB* mutants, respectively, compared to the control (Figure 3F). The biosynthesis of gliotoxin involves 13 genes within the cluster [17]. In both mutants, the expression of 13 genes (including TF GliZ) were upregulated to varying degrees (Figure 3F). Based on current understanding of the endocrocin biosynthetic pathway [42], four genes (*encA‐D*) in the cluster are involved. Transcriptome analysis revealed the upregulation of four genes by more than twofold in both mutants, with LaeB exerting more significant regulation than CsdA (Figure S8E). Conversely, all gene expressions in the neosartoricin and fumihopaside BGCs were significantly down‐regulated in both mutants (Figure S8F,G). Collectively, although CsdA/LaeB orchestrates multiple mycotoxins, FqC emerges as the predominant regulated metabolite.

### 2.5 FqC is the Effector of CsdA/LaeB‐Mediated Virulence

**FIGURE 4:**
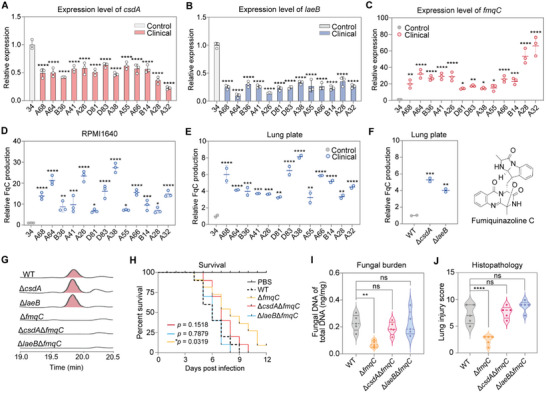
FqC is effector for CsdA/LaeB‐mediated virulence. A–C) The expression levels of *csdA* (A), *laeB* (B), and *fmqC* (C) in clinical strains compared to environmental strain 34. The expression levels of related genes in clinical strains were normalized to control strain 34. D,E) Comparative metabolomic analysis of FqC abundance between clinical and control strains in RPMI1640 (D) and lung plate (E). F) The FqC abundance of *A. fumigatus* and its mutants in lung plate medium. G) Metabolic profiles of *fmqC*‐associated deletion mutants. UV absorptions at 254 nm were illustrated. H) Survival curves of Balb/c mice intranasally infected with Δ*fmqC* single mutant, Δ*csdA*Δ*fmqC* or Δ*laeB*Δ*fmqC* double mutant compared with control strains (*n* = 10, log‐rank test). I) Fungal burden in the lungs 3 d post‐infection with Δ*fmqC* single mutant, Δ*csdA*Δ*fmqC* or Δ*laeB*Δ*fmqC* double mutant and control strains (*n* = 5). J) Histopathological assessment of lung injury on day 3 post infection with *A. fumigatus* and its mutant (*n* = 5). Quantification of lung tissue injury was performed according to the Smith scoring criteria. All error bars are expressed as mean ± SD. Statistical analysis was performed by using One‐way ANOVA (“ns”: Not significant. Significant at *p < 0.05, **p < 0.01, ***p < 0.001, ****p < 0.0001).

To further gain additional support for the regulation of secondary metabolism contributing to virulence in the *csdA* and *laeB* deletion mutants (Figure S10), we measured the expression levels of *csdA* and *laeB* as well as their co‐regulated BGC backbone genes by qRT‐PCR across clinical strains. The results showed that compared with the environmental strain 34, the clinical isolates had relatively lower mRNA expression levels of *csdA* and *laeB*, with *laeB* showing an even lower expression (Figure 4A,B). Interestingly, the backbone gene expression and production of FqC in multiple clinical strains were significantly higher than control group (*p* < 0.05), which is associated with the negative regulation of this metabolite by the CsdA and LaeB (Figure 4C,D).

We cultured clinical strains in mouse lung homogenate to identify CsdA‐LaeB‐associated pathogenic metabolites (Figure S11). Compared with environmental strain 34, the clinical isolates exhibited a more than two‐fold increase in FqC production (Figure 4E). To establish the correlation between FqC and CsdA‐LaeB, we compared FqC abundance in Δ*csdA* and Δ*laeB* mutants versus the control strain using lung plate medium. Quantitative analyses revealed that FqC production was up‐regulated 5.29‐ and 4.02‐ fold in the Δ*csdA* and Δ*laeB* mutants, respectively—aligning with clinical isolates where repressed *csdA*/*laeB* expression coincides with a surge in FqC levels (Figure 4F). Subsequently, a Δ*fmqC* mutant was constructed in the same CEA17 background (Figure S12), and its product and virulence compared to the Δ*csdA* mutant and wild‐type strains. The target product of the Δ*fmqC* mutant is absent (Figure 4G), accompanied by significantly attenuated virulence, with 60% mortality at 8 days’ post‐infection compared to 90% in wild‐type strain (*p* < 0.05, Figure 4H). Quantitative fungal burden analysis revealed reduced lung colonization in mice infected with Δ*fmqC* mutant compared with wild‐type strains (*p* < 0.001, Figure 4I). These findings suggest that CsdA/LaeB‐mediated virulence may be primarily attributable to its regulation on FqC biosynthesis.

To explore whether FqC serves as the primary metabolite underlying CsdA/LaeB‐induced virulence, we generated Δ*csdA*Δ*fmqC* and Δ*laeB*Δ*fmqC* double mutants in the CEA17 background (Figure S12) and compared their virulence to the Δ*fmqC* single mutant and wild‐type strains. Metabolic profiling revealed that neither the Δ*csdA*Δ*fmqC* and Δ*laeB*Δ*fmqC* double mutants nor the Δ*fmqC* single mutant produced FqC, compared to the control strain (Figure 4G). Critically, the mortality of Δ*csdA*Δ*fmqC* and Δ*laeB*Δ*fmqC* double mutants was restored to wild‐type levels, compared to that of the single mutants (Figure 4H). Furthermore, there were no significant differences in fungal colonization or histopathology between the double mutants and the control strains (*p* > 0.05, Figure 4I,J; Figure S13). These findings establish FqC as the effector of CsdA/LaeB‐mediated *A. fumigatus* virulence. To further validate this, we adjusted conidia of the Δ*csdA* and Δ*laeB* mutants to an infectious dose‐equivalent (2 × 10^6^ conidia), quantified FqC abundance, and calculated its relative content per conidium. Meanwhile, equal‐mass hyphal samples were collected for metabolite analysis. The results demonstrated that FqC abundance was significantly higher in conidia of both mutants compared to the control strain (Figure S14). These findings confirm that the enhanced virulence of the Δ*csdA* and Δ*laeB* mutants indeed stems from elevated FqC levels in the inoculated conidia.

## 3 Discussion

Although *A. fumigatus* virulence has been characterized at genomic and transcriptomic levels [3, 43], the contribution of global secondary metabolism to infection remains enigmatic. Fungal virulence has traditionally been attributed to thermotolerance [8, 44], cell wall modifications [45, 46], nutritional adaptability [47], biofilm [48], and stress responses [49, 50]. Here, our study unveils a conserved metabolic regulatory hub in *A. fumigatus* where the RNA‐binding protein CsdA partners with the global regulator LaeB to govern global secondary metabolism, and it mediates virulence primarily by repressing the biosynthesis of the pathogenic metabolite FqC. This discovery expands current models of fungal virulence regulation by defining a post‐transcriptional control layer that links RNA metabolism to secondary metabolism.

Since the biosynthetic genes of fumiquinazoline were identified in 2010 [51], this metabolite has been shown to localize to conidia and be regulated by the spore‐specific TF BrlA [52]. Previous studies identified the TF SebA as a negative regulator of FqC [32, 53, 54], but our work reveals a parallel regulatory mechanism involving CsdA‐LaeB, suggesting a novel post‐transcriptional control network for SM biosynthesis. In *P. fici*, CsdA exerts its functional by controlling alternative splicing of downstream genes to regulate their mRNA expression [27]. Our previous findings suggest that, across fungal pathogens, CsdA may act as a regulatory hub in virulence modulation—either through interacting with LaeB or coordinating secondary metabolism at the post‐transcriptional level. The conservation of the CsdA‐LaeB regulatory hub across fungal pathogens reveals an unprecedented metabolic‐virulence regulatory network, providing an alternative framework to dissect pathogenic mechanisms in diverse fungi. Notably, mining genomic data of clinical *A. fumigatus* [55] yielded CsdA/LaeB sequences from database (Table S5). Multiple sequence alignment revealed this regulatory hub exists not only in strains associated with invasive and chronic pulmonary infections, but also likely in drug‐resistant strains (Figure S15).

**FIGURE 5:**
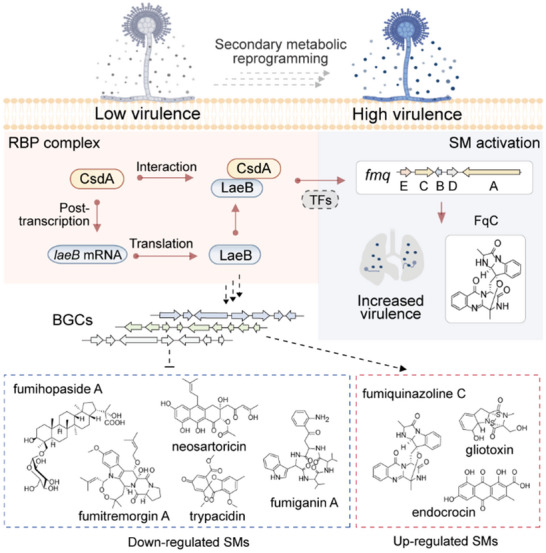
Model of CsdA‐LaeB interaction regulating FqC to drive *A. fumigatus* virulence. During pulmonary infection by *A. fumigatus*, repressed expression of the upstream CsdA‐LaeB proteins affects the transcription of distinct downstream transcription factors (TFs), thereby remodeling the biosynthesis of multiple secondary metabolites (SMs). Fumiquinazoline C (FqC), as a primary effector of CsdA/LaeB‐mediated virulence, drives fungal colonization and lethality in the lung tissue. Figure was created in BioRender. Song, Z. (2025) https://BioRender.com/qwjrk0k.

Pan‐metabolomics analysis of clinical and environmental isolates of *A. fumigatus* demonstrated differences in metabolic ion abundance between the two groups (Figure 1A,B). Notably, FqC was significantly enriched in clinical strains and regulated by CsdA and LaeB, suggesting its involvement in the CsdA/LaeB‐mediated virulence regulatory pathway (Figures 3E and 4D). This was subsequently demonstrated by constructing double mutants of Δ*csdA*Δ*fmqC* and Δ*laeB*Δ*fmqC* and in a murine model of invasive aspergillosis (Figure 4H–J). Genetic dissection revealed that CsdA and its nuclear interactor LaeB that orchestrates FqC biosynthesis and virulence by forming a CsdA‐LaeB‐FqC regulatory hub in *A. fumigatus* (Figure 5). Disruption of the protein interaction (Δ*csdA* or Δ*laeB*) triggered a 13.4‐ or 19.8‐ fold FqC surge (Figure 3E), correlating with a rise in murine mortality (up to 100% by day 7 post‐infection, Figures 1H and 2C). Furthermore, it will be important to investigate whether the CsdA‐LaeB interaction directly regulates transcription by binding to the promoters of key biosynthetic genes, for example, through chromatin immunoprecipitation (ChIP) assays.

The virulence increased phenotype of Δ*csdA* and Δ*laeB* mutants indicates that the CsdA‐LaeB interaction normally restrains FqC production to balance fungal virulence with host adaptation. Metabolites on the conidial surface typically play a role in immune recognition. FqC, localized on the surface of conidia, is likely to interfere with immune recognition, thereby facilitating conidial escape from host immune clearance. This mechanism—whereby CsdA‐LaeB regulates FqC synthesis to balance virulence—essentially reflects the environmental adaptive strategy of fungal conidia. Uncontrolled FqC production (e.g., when the CsdA‐LaeB interaction is non‐functional) leads to excessive accumulation, which may either increase the risk of conidial recognition by the host immune system or impose a heavy metabolic burden on the fungus (impairing its colonization efficiency in the host), disrupting the balance between “immune evasion and stable colonization.” The reduced expression of CsdA and LaeB in clinical isolates, coupled with elevated FqC, supports a role for this regulatory axis in pathogen evolution, potentially enabling *A. fumigatus* to fine‐tune virulence during adaptation to the host environment. Notably, environmental isolates may be evolving to repress the expression of the CsdA and LaeB proteins, thereby gaining a selective advantage during fungal infection.

From a disease diagnosis perspective, our findings highlight FqC as a potential metabolic biomarker for IA. The strong correlation between FqC production and virulence, coupled with its significant enrichment in clinical isolates, provides a foundation for developing diagnostic tools to distinguish pathogenic strains. To further solidify the role of FqC as a virulence determinant, future work could involve constructing strains that overexpress the FqC biosynthetic gene cluster to assess if this leads to a corresponding enhancement in pathogenicity. Mass spectrometry‐based FqC detection in bronchoalveolar lavage fluid could offer a rapid diagnostic approach [56, 57], particularly for immunocompromised patients at high risk for IA. Additionally, the CsdA‐LaeB‐FqC axis represents a vulnerable metabolic checkpoint for antifungal therapy. Small‐molecule inhibitors that restore CsdA‐LaeB interaction could potentially reactivate FqC suppression, thereby attenuating fungal virulence, and offering an alternative strategy for the treatment of fungal infections.

This study also raises several intriguing questions for future investigation. How does FqC specifically modulate host immune response in a neutropenic mouse model? What is the molecular basis of CsdA‐LaeB interaction during fungal infection, and can this interaction be pharmacologically targeted? How does RBP coordinates fungal metabolic virulence at the RNA metabolism level? Does the CsdA‐LaeB interaction regulate secondary metabolite synthesis through a direct or indirect mechanism? Answering these questions will not only deepen our understanding of fungal pathogenesis but also provide a framework for developing innovative therapeutic strategies against IA, a disease with pressing unmet medical needs.

In conclusion, we have defined a metabolic regulatory hub that coordinates *A. fumigatus* virulence through FqC biosynthesis. This work not only expands our mechanistic understanding of fungal pathogenesis but also provides a foundation for the development of novel diagnostic and therapeutic approaches. By uncovering the CsdA‐LaeB‐FqC axis, we have identified a metabolic checkpoint in fungal virulence regulation that holds promise for combating refractory IA and other invasive fungal diseases.

## 4 Experimental Section

### 4.1 Antibodies

Anti‐His, Anti‐GST, and HRP‐Goat Anti‐Mouse from Proteintech (66005‐1‐Ig, 66001‐2‐Ig, SA00001‐1) were used for western blot assays.

### 4.2 Mice

Balb/c mice were purchased from Beijing Vital River Laboratory Animal Technology Co., Ltd. Male mice (age, 7–8 weeks) were injected with cyclophosphamide and cortisone acetate to generate a murine model of invasive aspergillosis.

### 4.3 Ethics Statement

All murine experiments were performed in strict accordance with the “the regulation of the Institute of Microbiology, Chinese Academy of Sciences of Research Ethics Committee.” The murine experiment protocol was approved by the Institute of Microbiology, Chinese Academy of Sciences of Research Ethics Committee (Permit No. APIMCAS2022106).

### 4.4 Strains and Cultivation

The strains used in this study are listed in Table S6. All *Aspergillus fumigatus* strains were grown at 37°C on glucose minimum medium (GMM) with appropriate supplements corresponding to the auxotrophic marker or antibiotics [58, 59]. *A. fumigatus* and its transformants were cultivated in GMM medium at 25°C for 3 d to extract total RNAs and for 5 d to detect secondary metabolites (SMs). *Escherichia coli* strains DH5α and BL21 were cultured in LB medium (1% tryptone, 0.5% yeast extract, 1% NaCl) supplemented with appropriate antibiotics to construct plasmids and express proteins, respectively. *Saccharomyces cerevisiae* BJ5464 [60] were used for construction of green fluorescent protein (sfGFP) and red fluorescent protein (mCherry) expression vectors on synthetic dextrose complete medium with appropriate supplements corresponding to the auxotrophic markers [61].

### 4.5 Gene Cloning and Plasmid Construction

All plasmids and primers (Sangon Biotech Co. Ltd, China) are given in Table S6. For the construction of deletion mutants, around 1 kb upstream and downstream fragments of the targeted genes were amplified from *A. fumigatus* genomic DNA (gDNA) by high‐fidelity DNA polymerase TransStart *FastPfu* (Transgen Biotech, China). The marker genes *AfpyrG* and *hph* were amplified from the vectors pYH‐WA‐pyrG and pXW55‐hph, respectively, and fused with the flanking sequences of the target genes to form different deletion cassettes by the double‐joint PCR method described previously [62]. For protein expression, high‐fidelity DNA polymerase Q5 (New England Biolabs) was used to amplify the open reading frames (ORFs) of targeted genes that were then inserted into pET28a (*His_6_*‐tag) or pGEX‐4T (*GST*‐tag) to produce pYSZL46 (LaeB‐His) or pYSZL47 (GST‐CsdA) through the quick‐change method. For the subcellular localization of CsdA, the *csdA*, *mCherry*, and *hph* were integrated into the *Spe*I/*Pml*I‐cleaved pXW55 vector via the yeast recombination method [63] to give the plasmid pYSZL13 (*csdA‐mCherry*). The same method was used to construct *sfGFP* expression vectors pYSZL11 (*laeB‐sfGFP*). In order to construct complementary vectors, *csdA* and *hph* were integrated into the *Spe*I/*Pml*I‐cleaved pXW55 vector via the Clone Express MultiS One Step Cloning Kit (VAZYME BIOTECH Co. Ltd, CHINA) to give the plasmid pYSZL56 (*csdA‐hph*). The same method was used to construct pYSZL57 (*laeB‐hph*). The above plasmids were verified by PCR with 2 × T5 Super PCR Mix (Tsingke Biotechnology Co., Ltd, China).

To construct bimolecular fluorescence complementation (BiFC) vectors, the *AfpyrG* or *hph* gene was cloned into the *Kpn*I/*Hind*III‐cleaved pCX62‐CYFP or pKNT‐NYFP plasmid to give the empty vector pYSZL32 (*AfpyrG‐cyfp*) or pYSZL33 (*hph*‐*nyfp*) using the Clone Express MultiS One Step Cloning Kit, respectively. Each gene of *csdA* and *laeB* was fused with *gpdA* and integrated into pYSZL32 or pYSZL33 to obtain pYSZL25 or pYSZL28, respectively. Above plasmids were verified by sequencing (Sangon Biotech Co. Ltd, China).

### 4.6 Fungal Genetic Manipulations

The *csdA* or *laeB* gene in *A. fumigatus* was deleted according to the method described previously [59]. Briefly, the deletion cassette of *csdA* or *laeB* was transformed into *A. fumigatus* CEA17.2 (*AfpyrG*‐deficient) to produce single mutant TYYJ14 or TYSZL12, respectively. For the subcellular localization, the *laeB‐sfGFP‐AfpyrG* (7.1 kb) and *csdA‐mCherry‐hph* (6.9 kb) fragments were amplified from pYSZL11 and pYSZL13, respectively, and were transformed into *A. fumigatus* CEA17.2 and CEA17.1 (prototrophic strain) to produce strains TYSZL17 and TYSZL18. Meanwhile, the *laeB‐sfGFP‐AfpyrG* and *csdA‐mcherry‐hph* fragments were transformed together into *A. fumigatus* CEA17.2 to obtain the co‐localized strain TYSZL21. For the BiFC assays, each pair of constructed vectors (pYSZL28 and pYSZL32, pYSZL33 and pYSZL25, pYSZL28 and pYSZL25) were co‐transformed to *A. fumigatus* CEA17.2 to generate strains TYSZL37, TYSZL36, TYSZL25, respectively. The same method was used to construct TYLYX3 (Δ*fmqC‐hph*), TYSZL79 (Δ*fmqC‐hph*, Δ*laeB‐AfpyrG*) and TYSZL80 (Δ*fmqC‐hph*, Δ*csdA‐AfpyrG*). All the above transformants were verified by diagnostic PCR.

### 4.7 Phylogenetic Analysis of CsdA or LaeB

The amino acid sequences of CsdA and LaeB in *A. fumigatus* were obtained by multi‐alignment with CsdA from *P. fici* or LaeB from *A. nidulans*, respectively. CsdA and LaeB in *A. fumigatus* were used as the query for a BLAST analysis at the NCBI website (www.blast.ncbi.nlm.nih.gov/Blast.cgi). Amino acid sequences of CsdA and LaeB homologues from 94 and 192 species were downloaded from the NCBI database, aligned with MEGA7 software, and manually adjusted. Two phylogenetic trees were constructed by MEGA7 software, and clustering were performed by the neighbor‐joining method, while the other parameters were default. The fungi from Basidiomycota or Mucoromycota were regarded as the outgroups of CsdA or LaeB phylogram. The reliability of internal branch was evaluated with 1000 bootstrap resampling. The phylogram was modified and optimized via the Interactive Tree of Life website (ITOL, http://itol.embl.de/).

### 4.8 Fungal Growth and Metabolomics Analysis

*Aspergillus fumigatus* CEA17 (prototrophic strain) and its mutants were activated and cultivated on GMM medium at 37°C for 3 d. The conidia were collected with 0.1% Tween‐80 and counted by a hemocytometer. A 0.5 µL aliquot containing 1000 conidia was point‐inoculated onto GMM medium and cultured at 37°C for 4 d to analyze the growth rate and conidiation number of *A. fumigatus* CEA17 and its mutants. For metabolomic analysis, 1×10^7^ spores of *A. fumigatus* CEA17 and its mutants were inoculated into 20 mL liquid GMM medium at 25°C for 5 d with shaking at 200 rpm. Subsequently, the lung tissues from 6‐week‐old Balb/c mice were homogenized in 0.165  MOPS buffer and supplemented with 3% agar to obtain lung‐mimicking agar medium (lung plate). To mimic the in vivo environment of mice, 1000 conidia of each strain (Δ*csdA*, Δ*laeB*, clinical and environmental strains) were point‐incubated on solid RPMI 1640 and murine lung plate medium at 37°C for 5 d. Each experiment was conducted in at least three biological replicates.

To avoid potential loss of metabolites distributed in different cellular compartments or secreted into the medium, the entire culture (including conidia, hyphae, and medium) was collected and extracted with 20 mL of ethyl acetate prior to metabolomics analysis. After vacuum concentration to obtain the crude extracts, which were dissolved in 1 mL methanol (MeOH), and then analyzed by LC‐HRMS equipped with an ODS column (C18, 250 × 4.6 mm, Waters XTERRA, 5 µm) with a flow rate of 1 mL min^−1^. The MeOH and water with 0.1% (v/v) formic acid were used as the elution solvent, and linear gradient conditions were as follows: 10%–30% MeOH in 0–10 min, 30%–70% MeOH in 10–40 min, 70%‐90% MeOH in 40–50 min, 100% MeOH in 50.1–60 min, and 10% MeOH in 60.1–65 min. Clinical and environmental strains were analyzed with an Agilent 1200 LC/MSD SL (Santa‐Clara, USA) with a flow rate of 1 mL min^−1^. The acetonitrile and water with 0.1% (v/v) formic acid were used as the elution solvent, and linear gradient conditions were as follows: 5%–100% acetonitrile in 0–30 min, 100% acetonitrile in 30–35 min, 100%–5% acetonitrile in 35–35.1 min, and 5% acetonitrile in 35.1–40 min. The mass spectrum data were collected and converted into a format containing retention time, *m/z*, and ion peak density, respectively. Differentially regulated metabolites were screened with |log_2_foldchange|>1 and ‐log_10_ (*p*‐value)>1.3 as the threshold.

### 4.9 Animal Model of *A. fumigatus* Infection

Virulence of *A. fumigatus* Cea17.1 (WT), Δ*csdA*, Δ*laeB*, Δ*csdA^C^*, Δ*laeB^C^*, Δ*fmqC*, Δ*csdA*Δ*fmqC* and Δ*laeB*Δ*fmqC* strains were assessed in a murine invasive aspergillosis model. Briefly, male Balb/c mice (Beijing Vital River Laboratory Animal Technology Co., Ltd., China) weighing about 19–21 g were immunosuppressed via administration of cyclophosphamide by separate intraperitoneal injections, one at 3 d (200 mg kg^−1^ of body weight) and the other at 1 d (200 mg kg^−1^ of body weight) before infection. The second treatment includes administration of cortisone acetate at a dose of 250 mg kg^−1^ by separate subcutaneous injection at 1 before infection. Anesthetized mice (10 mice per fungal strain) were infected by nasal instillation of 20 µL of 1 × 10^8^ conidia mL^−1^ (day 0) and monitored three times daily for 12 days’ post‐infection. All surviving mice were sacrificed at day 12. Survival analysis was performed by Log‐rank (Mantel‐Cox) test. Each experiment was independently repeated three times, and one of the replicate results is shown in the figure.

### 4.10 Fungal Burden and Histopathological Analysis

Lung tissues from mice infected with *A. fumigatus* Cea17.1 or its mutants for 3 d were removed and freeze‐dried. Subsequently, the dry lung tissue was homogenized in a CTAB (cetyl trimethylammonium bromide, Sigma) extraction buffer (100 mm Tris‐HCl pH 7.5, 0.7 m NaCl, 10 mm EDTA, 1% CTAB, 1% β‐mercaptoethanol) to extract total gDNA as described previously [64]. Briefly, the mixed samples were cracked at 65°C for 30 min and then mixed with 1 mL chloroform and centrifuged at 1500 ×*g* for 10 min. All sample supernatants were added to isopropyl alcohol in equal volume and mixed gently. After centrifugation at 1500 ×*g* for 10 min, the precipitate was washed with 70% ethanol and dissolved with distilled water. 1 ng µL^−1^ of *A. fumigatus* gDNA was continuously diluted twofold to obtain 12 different concentrations for a standard curve. The *X* axis is the log_2_ value of the known standard concentrations, and *Y* axis is the *Ct* value of each standard. The above extracted gDNA samples were diluted to 20 ng µL^−1^, and *afks1* was used as the internal gene for qPCR quantification. The contents of *A. fumigatus* and its mutants in lung tissues were obtained according to the standard curve. Each experiment was conducted in five biological replicates.

Lungs removed from mice infected for 3 d were fixed with 10% formalin, and subsequently embedded in paraffin. Subsequently, consecutive slices with 4–6 µm in thickness were obtained and stained with hematoxylin‐eosin (HE) and periodic acid‐Schiff (PAS) for histopathological studies. Quantification of lung tissue injury was performed according to the Smith scoring criteria. Each experiment was conducted in five biological replicates.

### 4.11 Fluorescence Detection and BiFC Assays

For subcellular localization of CsdA or LaeB, an appropriate number of spores of TYSZL17 (*laeB‐sfGFP*), TYSZL18 (*csdA‐mCherry*), or TYSZL21 (*laeB‐sfGFP, csdA‐mCherry*) was inoculated into liquid GMM medium at 37°C and shaken at 200 rpm for 8–10 h. The mycelia collected by centrifugation were fixed in 10% formalin for 30 min and washed with distilled water and stained by DAPI solution (final concentration: 10 µg mL^−1^, BIOSHARP, CHINA) for 15 min. The fluorescent images were obtained with a Zeiss Axioplan 2 imaging system with the AxioCam MRm camera (Carl Zeiss Microscopy) and were processed with IMAGEJ2 software (NATIONAL INSTITUTES OF HEALTH). The in vivo interaction between CsdA and LaeB was confirmed by the NYFP‐ or CYFP‐ tagged BiFC strains. The fluorescent images were obtained and processed as described above.

### 4.12 Expression and Purification of Proteins

*Escherichia coli* BL21 cells transformed with pYSZL47 were incubated at 37°C in LB medium containing 50 µg mL^−1^ kanamycin until OD_600_ = 0.6 to express GST‐CsdA recombinant protein. Next, 0.5 mm isopropyl β‐D‐1‐thiogalactopyranoside (IPTG) was used to induce protein expression at 16°C for 20 h. The cells collected by centrifugation were lysed (50 mm Tris, 150 mm NaCl, 1 mm DTT, pH 7.3) by freeze‐thaw, and debris was removed. Recombinant GST‐CsdA protein was purified with GST‐tagged resin (Beyotime, China) and eluted by elution buffer (50 mm Tris, 150 mm NaCl, 10 mm GSH, pH 8.0). The expression and purification of LaeB was as described above, but a different lysis buffer was used (50 mm NaH_2_PO_4_·2H_2_O, 300 mm NaCl, 10 mm imidazole, pH 8), wash buffer (40 mm imidazole), elution buffer (100 mm imidazole) and Ni‐NTA resin (QIAGEN, CA). Target proteins were detected and quantified by 12% SDS‐PAGE and Nano‐Drop C2000 (Thermo Fisher Scientific), respectively.

### 4.13 Pull‐Down and Western Blotting

The interaction between CsdA and LaeB was confirmed by pull‐down assays in vitro. Briefly, individual GST‐CsdA or LaeB‐His protein was incubated with Ni‐NTA resin at 4°C for 4 h in binding buffer. Both samples were centrifuged for 1 min at 4°C and 800 ×*g*, and the supernatant of CsdA was added to the precipitate of LaeB and incubated at 4°C overnight. Next, the above mixture was centrifuged for 1 min at 4°C and 800 ×*g*, and the precipitation was washed three times through the wash buffer. The CsdA‐LaeB‐resin mixture was denatured by heating, separated on 7.5% SDS‐PAGE, and then transferred to polyvinylidene fluoride (PVDF) membrane (PALL, USA). The CsdA and LaeB protein was detected with mouse monoclonal antibodies anti‐GST (Proteintech, 1:7000 dilution) and anti‐His (Proteintech, 1:10 000 dilution), respectively. HRP Goat‐Anti‐Mouse IgG (Proteintech, 1:5000 dilution) was used to hybridize with anti‐His or GST antibodies, respectively, and target bands were detected by ECL (Thermo, MA, USA).

### 4.14 Transcriptional Analysis by RNA‐seq and qRT‐PCR

Total RNAs from the mycelia of *A. fumigatus* CEA17.1, Δ*csdA* and Δ*laeB* strains were isolated using TriZol reagent (CATALOG NO. R1000, LABLEAD Inc., CHINA), and then the quality of RNA was evaluated by Agilent 2100 bioanalyzer. Total RNA examples were sequenced on Illumina NovaSeq 6000 (Illumina, USA) at Novogene Biotech Co. Ltd. Data quality was controlled by fastp (version 0.19.7) [65] based on sequencing error rate for a single base less than 1%. The clean reads were mapped to the reference sequence and visualized by HISAT2 [66] or Integrative Genomics Viewer (IGV) software, respectively. A total of 11,463 unique transcripts were detected in *A. fumigatus* and its mutants. Gene expression levels were represented using normalized FPKM (fragments per kilobase of transcript per million mapped reads). The differentially expressed genes were identified with *p* value and log_2_foldchange/ratio between *A. fumigatus* and mutants by DESeq2 [67]. Three biological replicates were performed for each strain.

Total RNAs of *A. fumigatus* Cea17.1, Δ*laeB* and Δ*csdA* were reverse transcribed into cDNA with an *Evo M‐MLV* Plus first Strand cDNA Synthesis Kit (Accurate Biotechnology(Hunan) Co. Ltd, Changsha, China) for quantitative real‐time PCR (qRT‐PCR) assays according to the manufacture's protocol. Briefly, qRT‐PCR was conducted using a KAPA SYBR FAST qPCR Kit (Kapa Biosystems, USA). The reaction including 2 × KAPA SYBR FAST qPCR Master Mix, 0.2 µM forward/reverse primer, about 2 µg cDNA template was carried out at 95°C for 3 min, followed by 40 cycles of (95°C for 3 s, 60°C for 20 s, 72°C for 20 s). Each cDNA sample was performed in triplicate, and relative expression levels were calculated using the 2^−ΔΔCt^ method. Using the same method, total RNA was extracted from clinical and environmental strains, and the relative expression levels of the *csdA*, *laeB*, and *fmqC* were determined via qRT‐PCR assays. Three biological replicates were performed for each strain.

### 4.15 Measurement of Splicing Ratio

The splicing rate of *laeB* and *fmqA‐E* introns in Δ*csdA* mutant were measured and compared with control strain by qRT‐PCR. Briefly, the splicing ratio was calculated by normalizing the spliced RNA abundance to the unspliced RNA abundance of each intron using the equation 2^Δ(Ct‐unspliced – Ct‐spliced)^. The forward and reverse primers of spliced RNA amplification were designed at cross exon‐exon junctions and inside the adjacent exon, respectively, but the forward and reverse primers of unspliced RNA amplification were designed in the intron region and inside the adjacent exon. Each experiment was performed independently with three biological replicates.

### 4.16 Quantification of FqC

The extraction of FqC was carried out according to the same method described above. Briefly, *A. fumigatus* was cultured on GMM or simulated medium and extracted by equal volume ethyl acetate, dried by vacuum, and then re‐suspended with 1 mL MeOH for HPLC analysis. Subsequently, FqC was separated on a Waters HPLC system (Waters e2695, Waters 2998, Photodiode Array Detector) using an ODS column (C18, 250×4.6 mm, Waters XTERRA, 5 µm) with a flow rate of 1 mL min^−1^. The methanol (A) and water with 0.1% (v/v) formic acid (D) were used as the solvent. Elution conditions were as follows: 0 to 30 min, 20% to 100% A; 30 to 35 min, 100% A; 35 to 35.1 min, 100% to 20% A. UV absorptions at 254 nm were illustrated. The peaks of FqC in the crude extracts were determined according to the retention time and molecular weight of the standard. Subsequently, the relative production of FqC was calculated according to its peak area by normalized to the control group. At least two replicates were performed for each strain.

### 4.17 Statistical Analysis

Statistical parameters and sample size (*n*) are shown in the corresponding figure legends. All statistical analyses were done in GraphPad Prism8 software. Quantification data are generally presented as bar/line plots, with the error bar representing mean ± SD. Asterisks were used to indicate statistical significance, *stands for *p* < 0.05; **p < 0.01, ***p < 0.001, and ****p < 0.0001.

## Author Contributions

W.‐B.Y. performed the conceptualization, supervision, funding acquisition. W.‐B.Y., Z.S., and H.Z. contributed to all stages of manuscript preparation and editing. Z.S. performed fungal phenotypic analysis, HPLC analysis, transcriptome and metabolome analysis, phylogenetic analysis, subcellular localization, pull‐down assay, BiFC assay and murine experiments. H.Z. and Y.L. performed the construction, fermentation, SMs extraction and qRT‐PCR experiments of fungal mutants. L.Y. assisted in constructing the infection model. L.W. supervised the murine infection experiments. X.L., C.Z., and K.H.W. analyzed and evaluated the data. N.M.M.S., H.L., and L.C. participated in the discussion of the results. N.P.K., B.R.O., and M.B. revised the manuscript. All authors have seen and approved the manuscript.

## Funding

This work was supported by the Chinese Academy of Sciences Project for Young Scientists in Basic Research and the Strategic Priority Research Program [Grant Nos. YSBR‐111 and XDB0830000]; the National Natural Science Foundation of China [Grant No. 32470046]; the National Key Research and Development Program of China [Grant No. 2022YFC2303000].

## Conflicts of Interest

The authors declare no conflicts of interest.

## Supporting information




**Supporting File 1**: advs73737‐sup‐0001‐SuppMat.pdf.

**Supporting File 2**: advs73737‐sup‐0002‐TableS1.xlsx.

**Supporting File 3**: advs73737‐sup‐0003‐TableS2.xlsx.

**Supporting File 4**: advs73737‐sup‐0004‐TableS3.xlsx.

**Supporting File 5**: advs73737‐sup‐0005‐TableS4.xlsx.

**Supporting File 6**: advs73737‐sup‐0006‐TableS5.xlsx.

**Supporting File 7**: advs73737‐sup‐0007‐TableS6.xlsx.

## Data Availability

RNA‐seq data generated in this study have been deposited in the Gene Expression Omnibus (GEO) database under accession code GSE312095. Other relevant data supporting the findings of this study are available in this article and its Supplementary Information files.
