# Supplementary material for: CsdA‐LaeB Regulatory Hub Contributes to Aspergillus fumigatus Virulence via Fumiquinazoline C Biosynthesis
Source: Adv Sci (Weinh). 2026 Jan 7;13(16):e19021. doi: 10.1002/advs.202519021 (PMC13042810; doi:10.1002/advs.202519021)
Supplement: Supplementary file 1 — Supporting File 1: advs73737‐sup‐0001‐SuppMat.pdf. [file ADVS-13-e19021-s001.pdf]

Supporting Information

CsdA-LaeB Regulatory Hub Contributes to *Aspergillus fumigatus* Virulence via Fumiquinazoline C Biosynthesis

*Zili Song, Hongjiao Zhang, Leixin Ye, Yuxin Lei, Linqi Wang, Xiao Liu, Nayanna M. Mercado Soto, Nancy P. Keller, Berl R. Oakley, Can Zhao, Michael Bromley, Hongwei Liu, Lei Cai, Koon Ho Wong, Wen-Bing Yin**

*Corresponding author: yinwb@im.ac.cn

This file includes:

Figure S1: Phylogenetic tree of RNA binding protein CsdA in pathogenic fungi.
Figure S2: Pulmonary pathological observations on day 3 post-infected *A. fumigatus* and Δ*csdA* mutant.
Figure S3: Radial growth and conidial production of *csdA* and *laeB* mutants compared to the control strain.
Figure S4: Pulmonary pathological observations on day 3 post-infected *A. fumigatus* and Δ*laeB* mutant.
Figure S5: I*n vitro* purification and pull-down workflow for CsdA or LaeB.
Figure S6: The splicing efficiency of *laeB* and *fmq* cluster introns in Δ*csdA* mutant were determined by qRT-PCR (*n* = 3).
Figure S7: Integrated transcriptomic-metabolomic analysis framework of CsdA- or LaeB-mediated global secondary metabolic change in *A. fumigatus*.
Figure S8: Regulatory network of the BGC genes regulated by CsdA and LaeB in *A. fumigatus*.
Figure S9: Transcriptomic analysis of gene expression of extragenic cluster transcription factors co-regulated by CsdA and LaeB in *A. fumigatus*.
Figure S10: Pipeline for identifying CsdA/LaeB-regulated virulence-associated metabolites.
Figure S11: The fumiquinazoline C abundance in clinical *A. fumigatus* cultured in lung plates.
Figure S12: Construction and metabolite analysis of backbone gene mutants related to fumiquinazoline C biosynthesis.
Figure S13: Pulmonary pathological observations on day 3 post-infected *A. fumigatus* and *fmqC*-related mutants.
Figure S14: HPLC analysis of fumiquinazoline C production in conidia and hyphae of Δ*csdA* and Δ*laeB* mutants compared to the control strain.
Figure S15: The prevalence of the CsdA-LaeB proteins in reported clinical *A. fumigatus* isolates.
Legends for Supplementary Table S1 to S6

Additional files:

Supplementary Table S1 to S6.

Supplementary Figures

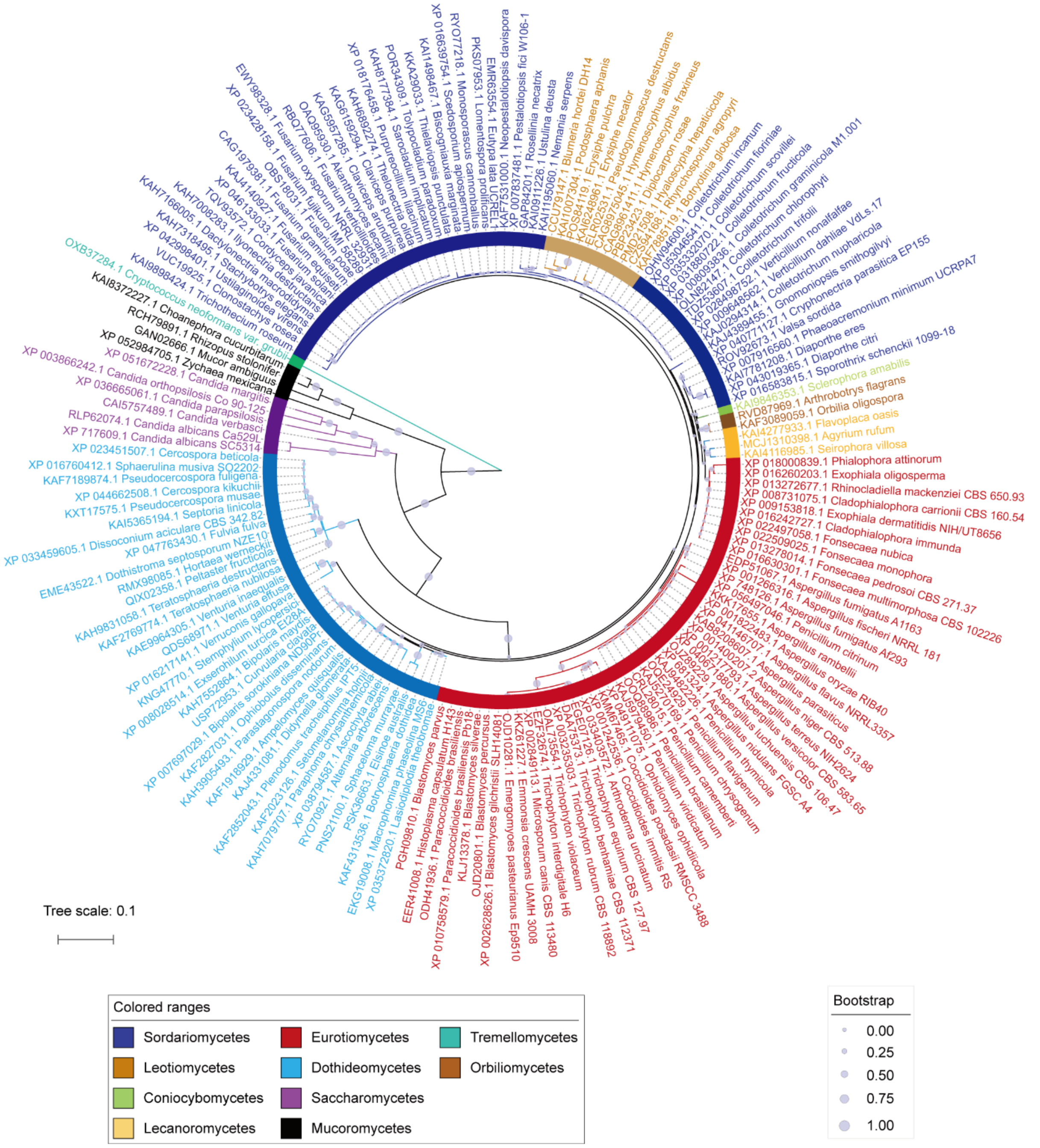

Figure S1. Phylogenetic tree of RNA binding protein CsdA in pathogenic fungi. Different colored circles represent taxonomic units of class level. Basidiomycete fungi (*Cryptococcus neoformans var. grubii*) was considered as outgroup. The homologues had more than 30% identity and more than 70% coverage. Related to Table S2.

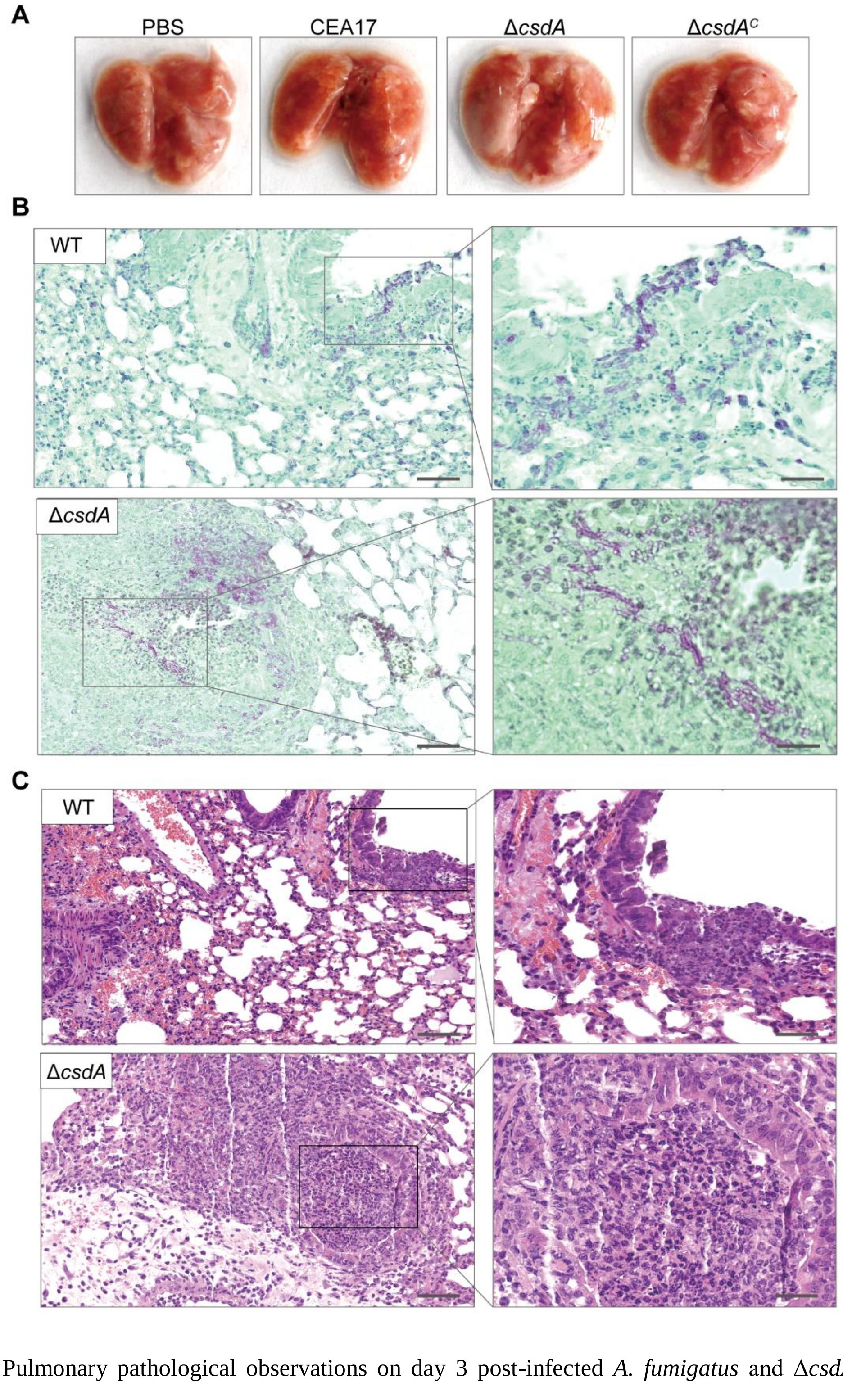

Figure S2. Pulmonary pathological observations on day 3 post-infected *A. fumigatus* and Δ*csdA* mutant. **A**) Macroscopic pathology of the lung collected on day 3 post-infected by *A. fumigatus* and Δ*csdA* mutant. **B**-**C**) Periodic Acid-Schiff (**B**) and Haematoxylin & Eosin (**C**) staining on day 3 post infection with *A. fumigatus* and its mutant. Scale bars, 100 μm (left panels). The right panels show the figure with a 3× local magnification.

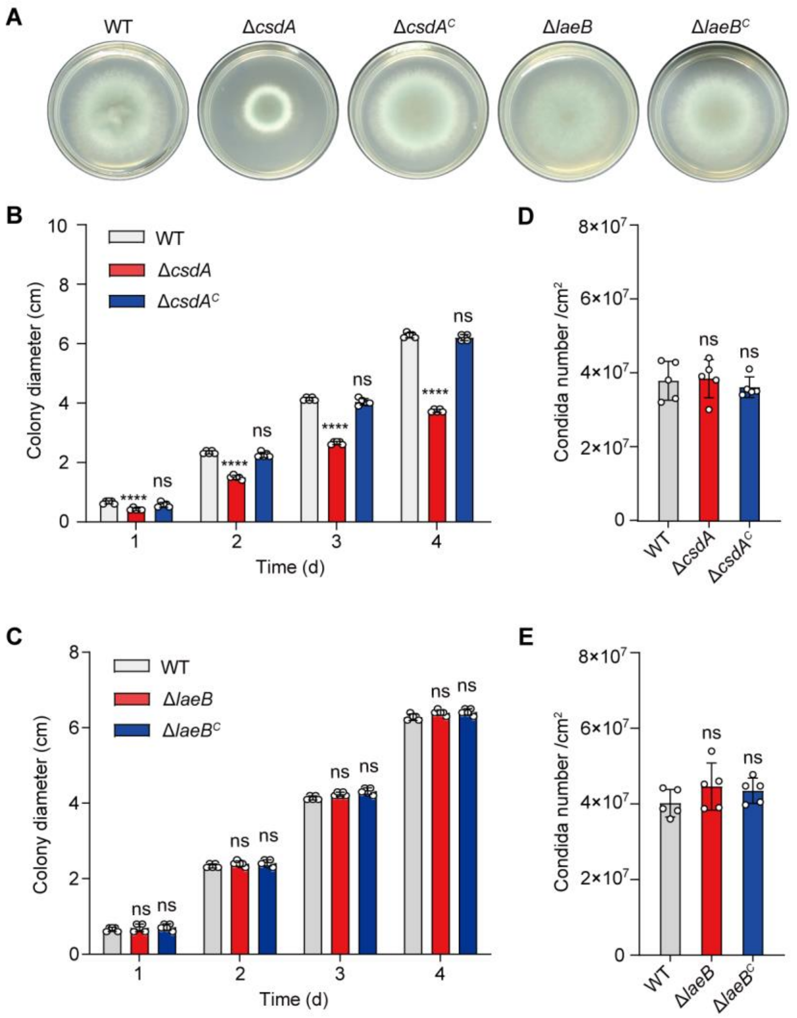

Figure S3. Radial growth and conidial production of *csdA* and *laeB* mutants compared to the control strain. **A**) Colony phenotypes of *A. fumigatus* wild-type and mutant strains grown on GMM medium at 37 ℃ for 3 days. **B-C**) Measurement of radial growth in the *csdA* (**B**) or *laeB* (**C**) deletion and complementation strains of *A. fumigatus* (n = 5). Statistical analysis was performed by using Two-way ANOVA ("ns": not significant. Significant at ****p < 0.0001). **D-E**) Conidial production in the *csdA* (**D**) or *laeB* (**E**) deletion and complementation strains versus the control of *A. fumigatus* (n = 5). Statistical analysis was performed by using One-way ANOVA ("ns": not significant). All error bars are expressed as mean ± SD.

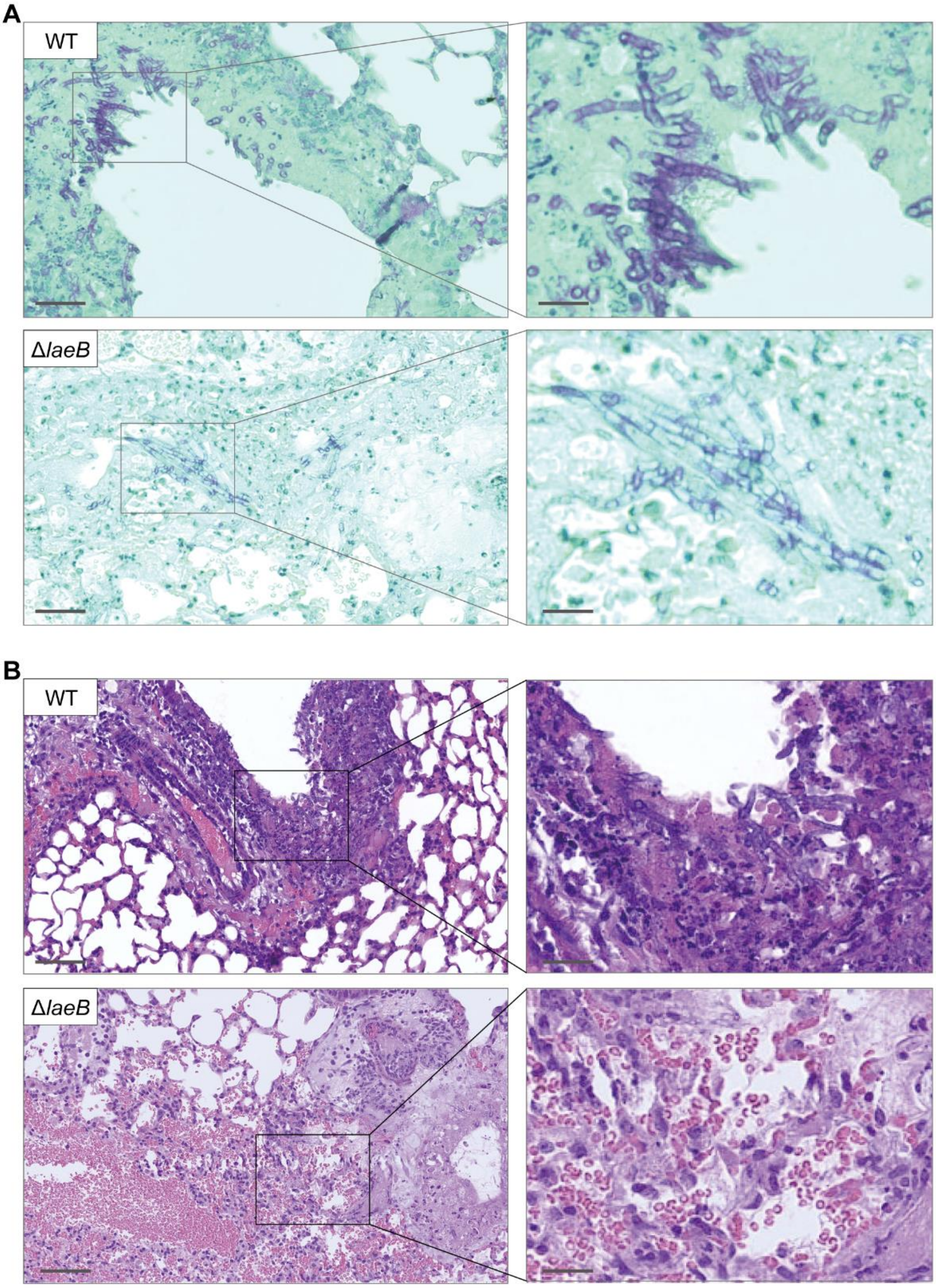

Figure S4. Pulmonary pathological observations on day 3 post-infected *A. fumigatus* and Δ*laeB* mutant. **A-B**) Periodic Acid-Schiff (**A**) and Haematoxylin & Eosin (**B**) staining on day 3 post infection with *A. fumigatus* and Δ*laeB* mutant. Scale bars, 100 μm (left panels). The right panels show the figure with a 3.5× local magnification.

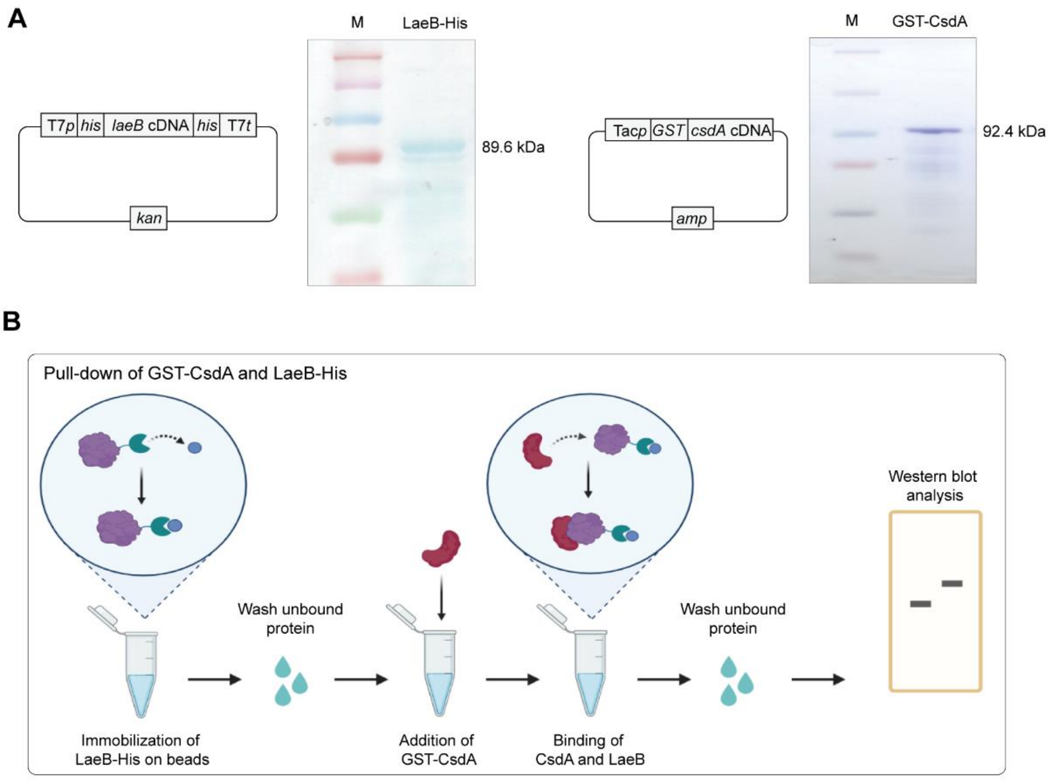

Figure S5. I*n vitro* purification and pull-down workflow for CsdA or LaeB. **A**) Brilliant blue G-stained 12% SDS-PAGE of LaeB or CsdA protein. M: marker. **B**) Schematic of CsdA and LaeB *in vitro* pull-down. Figure S5B was created in BioRender. Song, Z. (2025) https://BioRender.com/um0bl6f.

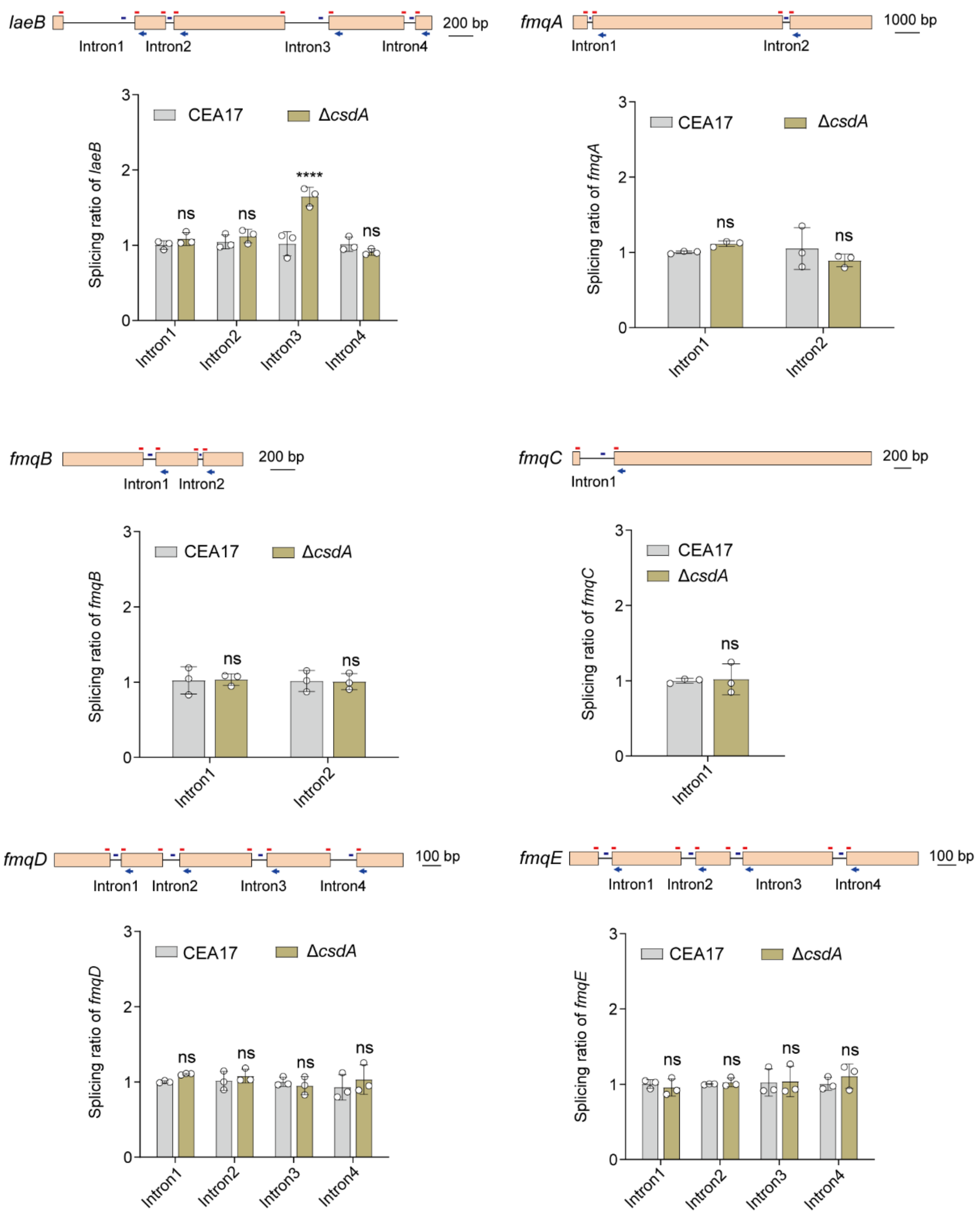

Figure S6. The splicing efficiency of *laeB* and *fmq* cluster introns in Δ*csdA* mutant were determined by qRT-PCR ($n = 3$). Deletion of *csdA* enhanced the splicing of intron 3 in *laeB* pre-mRNA, confirming its function as an RNA-binding protein. However, *csdA* deletion had no impact on the alternative splicing of genes within the *fmq* cluster, indicating it regulates fumiquinazoline C (FqC) biosynthesis indirectly via downstream genes. The splicing efficiencies were calculated as spliced RNA normalized to the level of unspliced RNA. A schematic of the genes is shown at the top. The red dashes in the diagram represent the primers designed to amplify regions composed of exon-exon junctions after intron removal (splicing), while the blue dashes represent the primers designed within the introns that remain unspliced. All error bars are expressed as mean ± SD. Statistical analysis was performed by using Two-way ANOVA (“ns”: not significant. Significant at ****$p < 0.0001$)

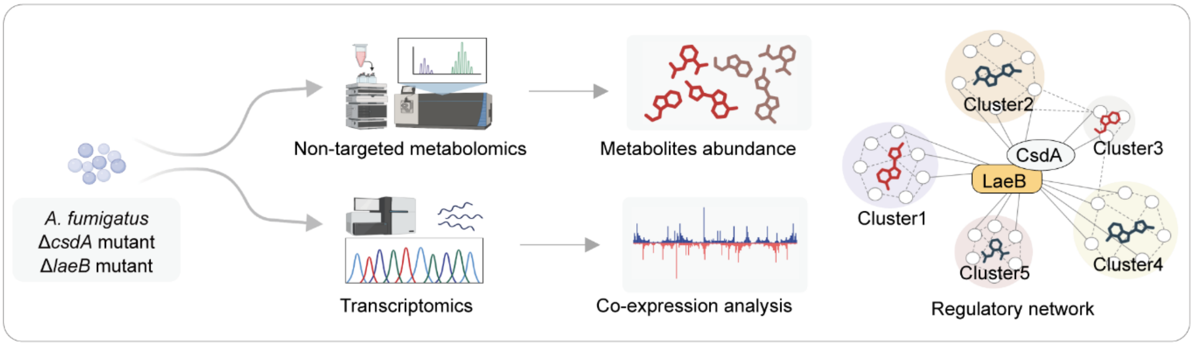

Figure S7. Integrated transcriptomic-metabolomic analysis framework of CsdA- or LaeB-mediated global secondary metabolic change in *A. fumigatus*. Figure was created in BioRender. Song, Z. (2025) https://BioRender.com/ltomcwa.

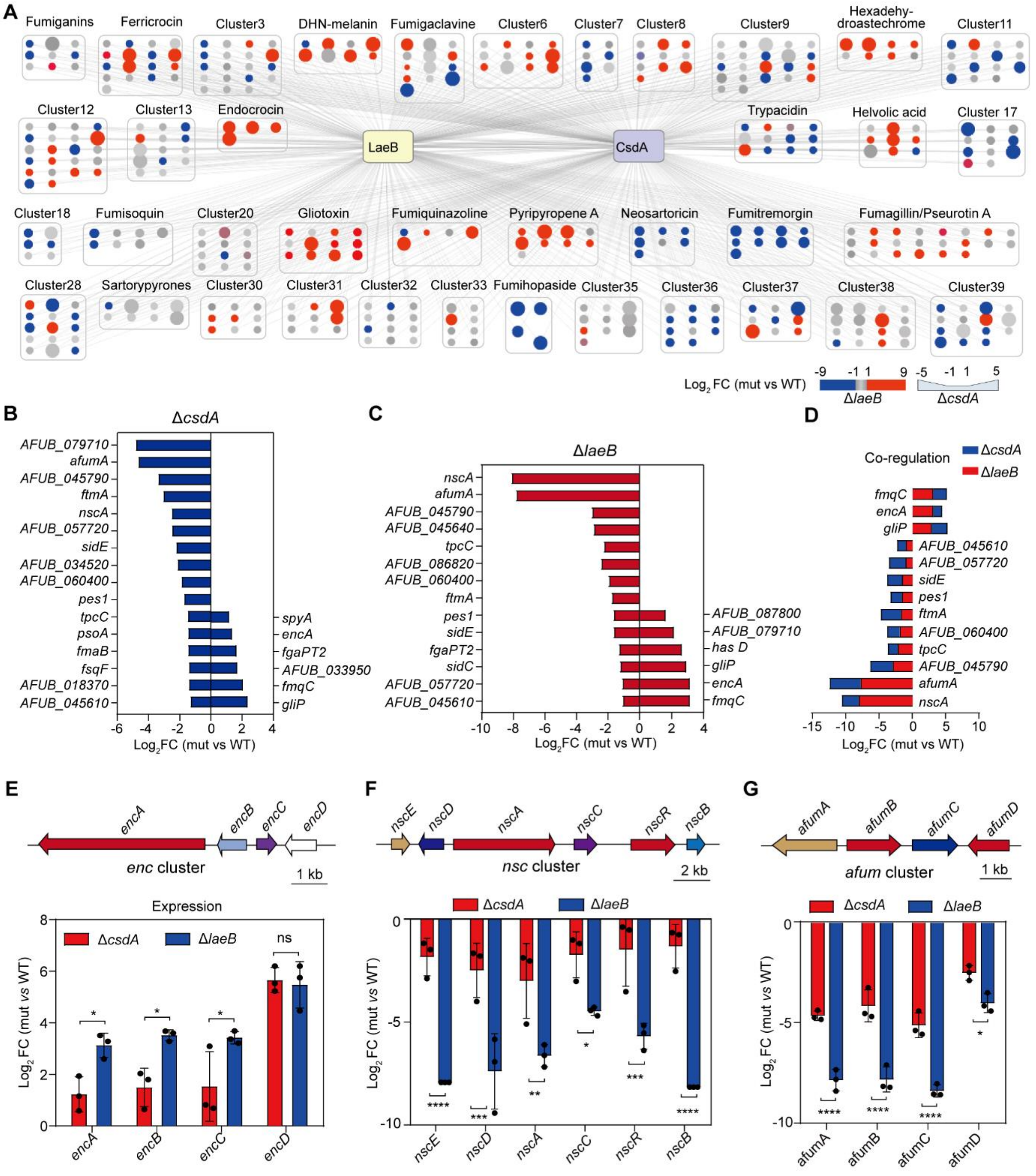

Figure S8. Regulatory network of the BGC genes regulated by CsdA and LaeB in *A. fumigatus*. **A**) Regulatory network of global secondary metabolism mediated by CsdA and LaeB in *A. fumigatus*. The gene expression levels of 39 BGC genes regulated by CsdA or LaeB in *A. fumigatus*. Red and blue dots indicate the genes that are significantly up-regulated and down-regulated by LaeB, respectively. The size of the dots represents the genes that are regulated by CsdA. **B-C**) The BGC backbone genes significantly regulated by CsdA or LaeB in *A. fumigatus*. **D**) The BGC backbone genes significantly co-regulated by CsdA and LaeB in *A. fumigatus*. **E**) The *enc* cluster gene expression in Δ*csdA* and Δ*laeB* mutants compared with control. The *enc* A-D genes are required for endocrocin biosynthesis. **F**) The *nsc* cluster gene expression in Δ*csdA* and Δ*laeB* mutants compared with control. The *nsc* A-E, R genes are required for neosartoricin biosynthesis. **G**) The *afum* cluster gene expression in Δ*csdA* and Δ*laeB* mutants compared with control. The *afum* A-D genes are required for fumihopaside biosynthesis.

Differentially expressed genes: $p<0.05$, $|Log_2foldchange|>1$. All error bars are expressed as mean ± SD. Statistical analysis was performed by using Two-way ANOVA ("ns": not significant. Significant at *$p < 0.05$, **$p < 0.01$, ***$p < 0.001$, ****$p < 0.0001$).

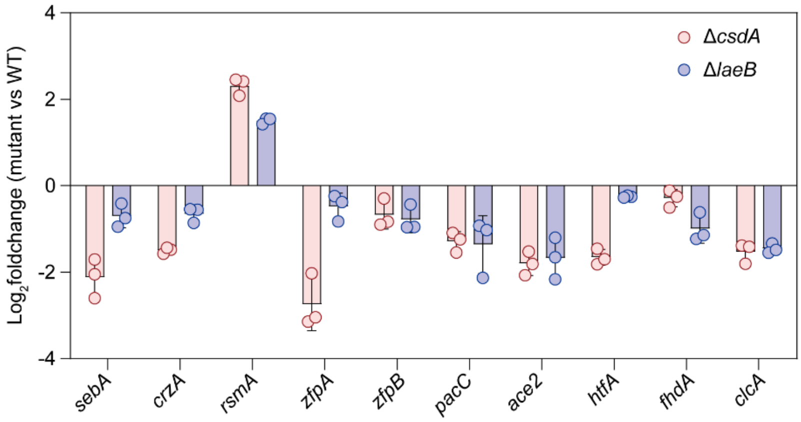

Figure S9. Transcriptomic analysis of gene expression of extragenic cluster transcription factors co-regulated by CsdA and LaeB in *A. fumigatus*. Multiple extragenic cluster transcription factors are co-regulated by CsdA and LaeB. All error bars are expressed as mean ± SD.

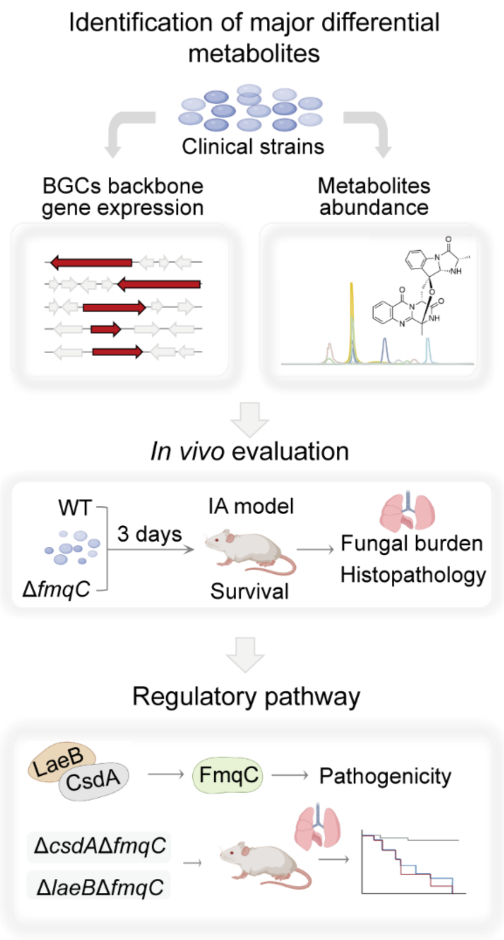

Figure S10. Pipeline for identifying CsdA/LaeB-regulated virulence-associated metabolites. Figure was created in BioRender. Song, Z. (2025) https://BioRender.com/5gfe20g.

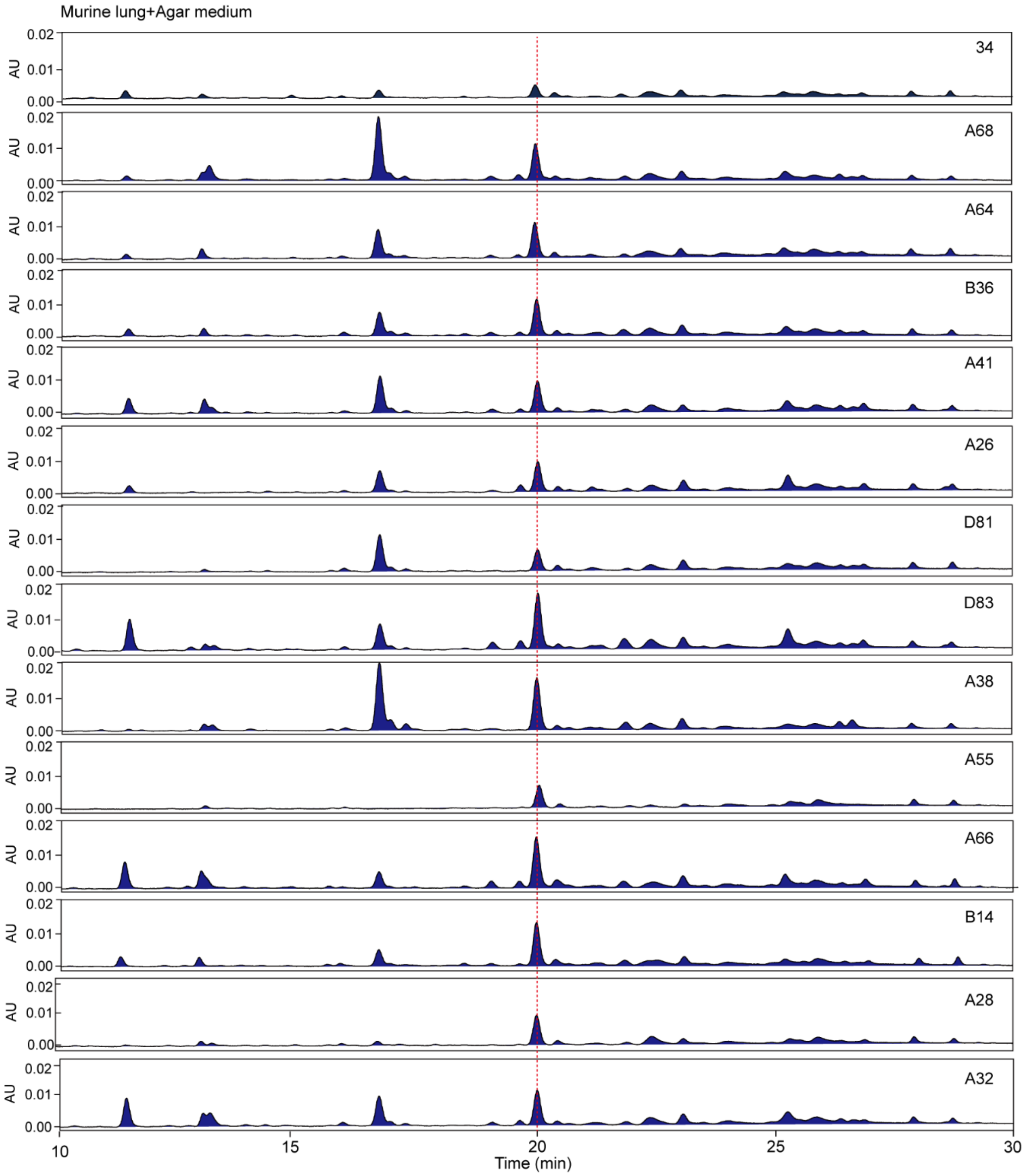

Figure S11. The fumiquinazoline C abundance in clinical *A. fumigatus* cultured in lung plates. Metabolic profiles of clinical strains cultured in lung plates. The red line represents the peak of fumiquinazoline C.

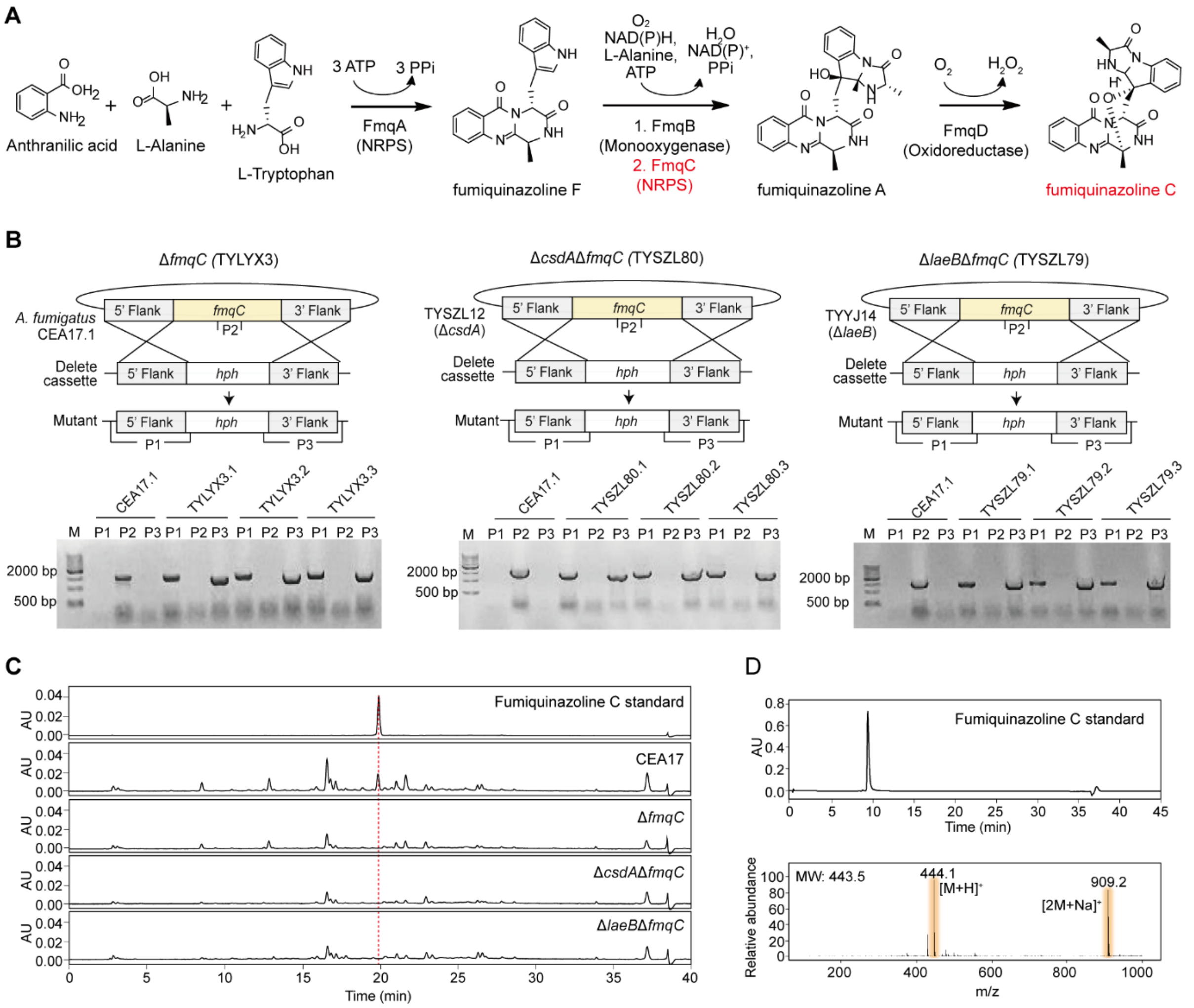

Figure S12. Construction and metabolite analysis of backbone gene mutants related to fumiquinazoline C biosynthesis. **A**) Biosynthetic pathway of fumiquinazoline C. NRPS: non-ribosomal peptide synthase. The *fmqA* and *fmqC* are the backbone genes in the biosynthetic pathway of fumiquinazoline C. **B**) Construction of the Δ*fmqC* single mutant or Δ*csdA*Δ*fmqC* and Δ*laeB*Δ*fmqC* double mutants by homologous recombination. The *hph* represents hygromycin resistance gene. P1 and P3 represent the primers for screening of the corresponding mutants. The specific bands (about 1.5 kb) were detected in mutants using the corresponding primers but not in control strain. The *fmqC* gene was detected in control using P2 primers, but not in the mutant strains. **C**) Metabolic profile of *fmqC*-related mutants. The red line represents the peak of fumiquinazoline C in the metabolic profile. **D**) The molecular weight of fumiquinazoline C was analyzed by LC-MS. MW: molecular weight.

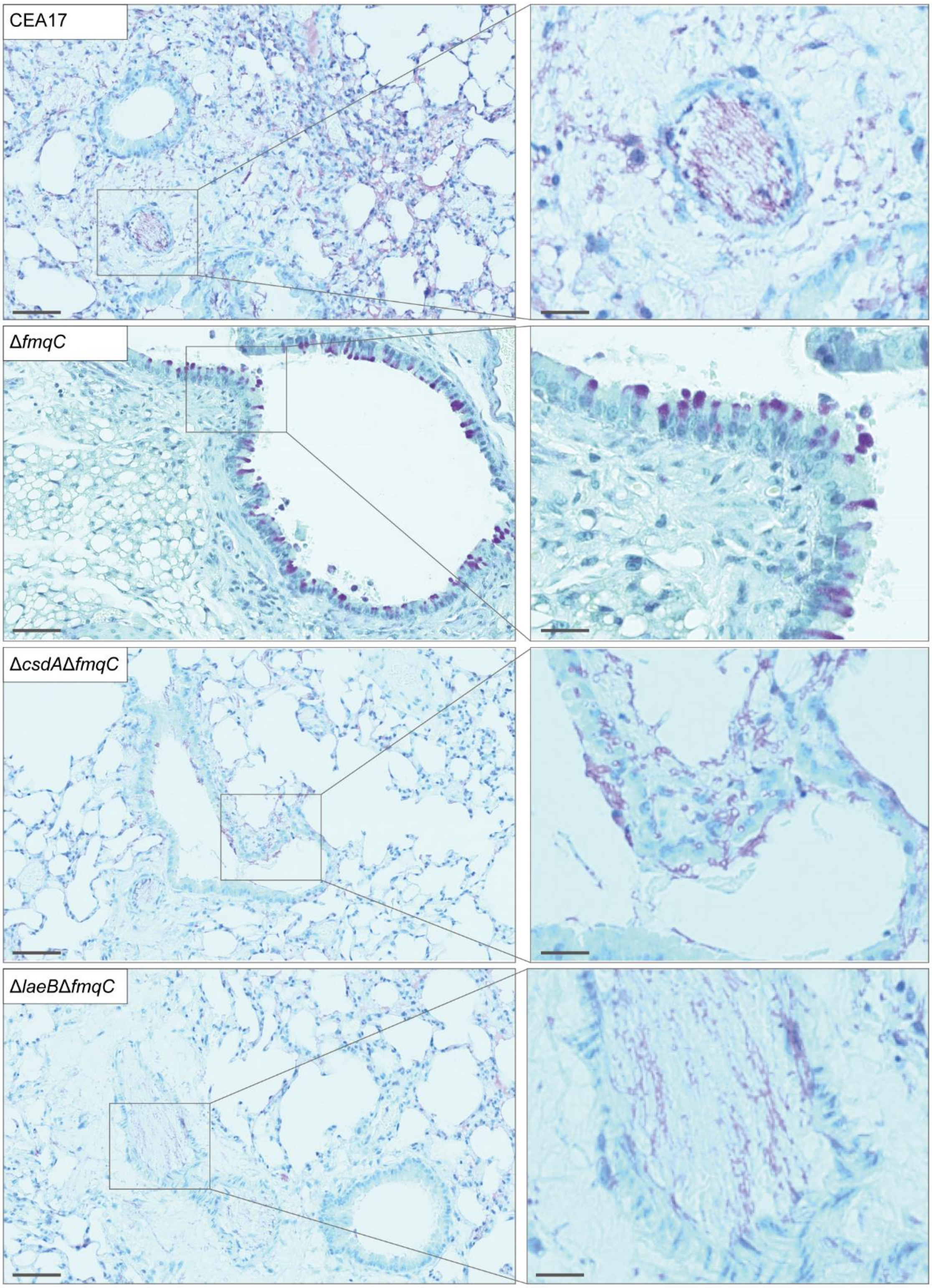

Figure S13. Pulmonary pathological observations on day 3 post-infected *A. fumigatus* and *fmqC*-related mutants. Periodic Acid-Schiff staining on day 3 post infection with *A. fumigatus* and *fmqC*-related mutants. Scale bars, 100 μm (left panels). The right panels show the figure with a 4× local magnification.

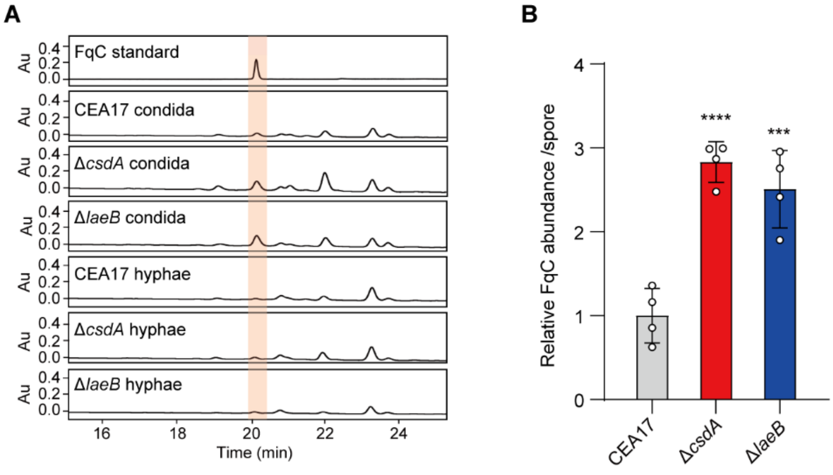

Figure S14. HPLC analysis of fumiquinazoline C production in conidia and hyphae of Δ*csdA* and Δ*laeB* mutants compared to the control strain. **A**) HPLC analysis of fumiquinazoline C (FqC) production in hyphae and conidia of the Δ*csdA* and Δ*laeB* mutants was performed at a wavelength of 254 nm. **B**) The relative content per conidium was calculated based on the fumiquinazoline C abundance in the metabolic profiles of the Δ*csdA* and Δ*laeB* mutants (n = 4). All error bars are expressed as mean ± SD. Statistical analysis was performed by using One-way ANOVA (Significant at ***p < 0.001, ****p < 0.0001).

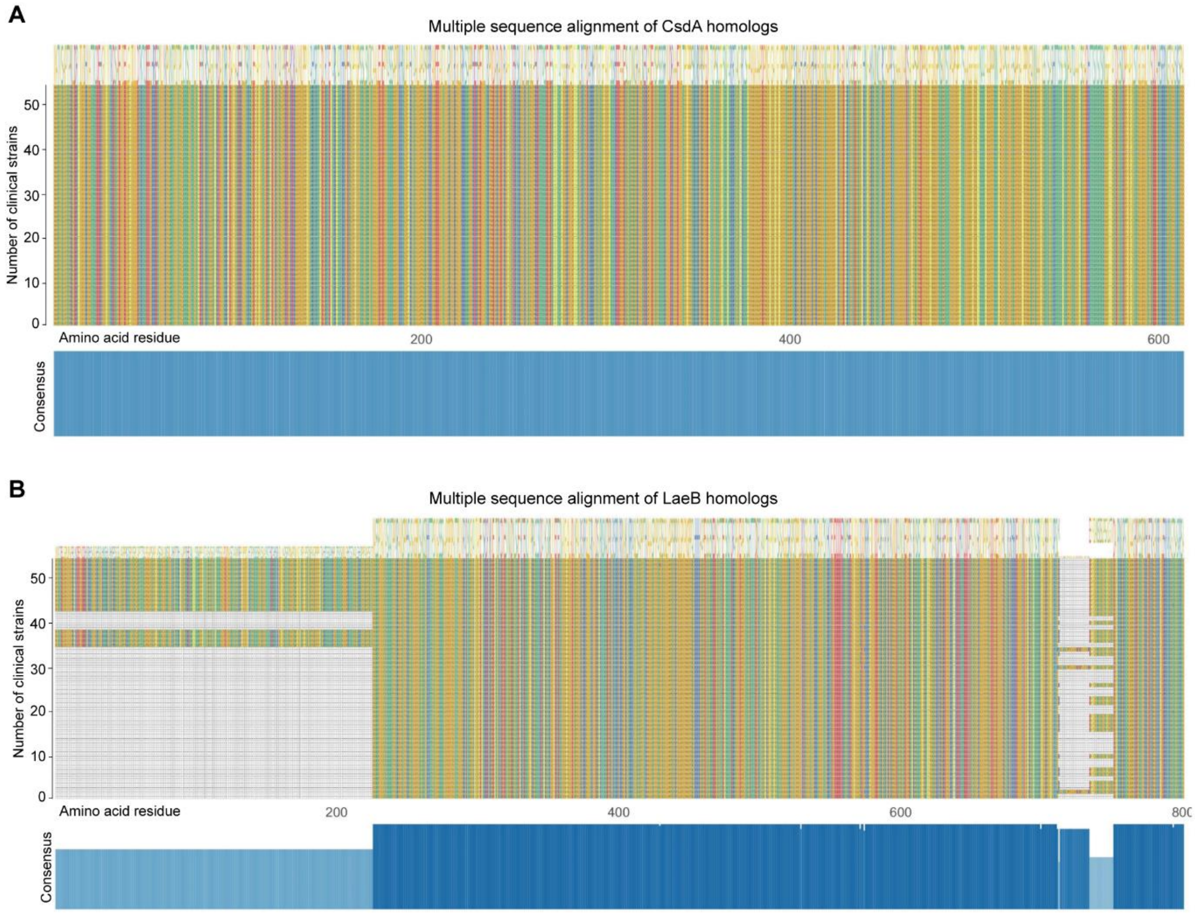

Figure S15. The prevalence of the CsdA-LaeB proteins in reported clinical *A. fumigatus* isolates. **A**) Multiple sequence alignment of the CsdA protein among 54 reported clinical *A. fumigatus* isolates. **B**) Multiple sequence alignment of the LaeB protein among 54 reported clinical *A. fumigatus* isolates. All strain information was provided in Table S5.

Supplementary Table

Table S1. Mass spectrometry data used for clinical *Aspergillus fumigatus* metabolome analysis. Sheet 1: Total metabolic ion products detected in clinical and environmental *A. fumigatus* isolates. Sheet 2: Differential metabolite production in clinical *A. fumigatus* compared to environmental strain 34.

Table S2. Species information used to construct phylogenetic trees. Sheet 1: Species containing CsdA homologues. Sheet 2: Species containing LaeB homologues.

Table S3. High-resolution mass spectrometry data of *A. fumigatus* mutant strains. Sheet 1: Total metabolic ion products detected in *A. fumigatus* and its single mutants. Sheet 2: Regulated metabolic ion products in Δ*csdA* mutant compared with control. Sheet 3: Regulated metabolic ion products in Δ*laeB* mutant compared with control. Statistical analysis was performed by using *t* test (two-tailed). Differential metabolic ions: $p<0.05$, $|\text{Log}_2\text{foldchange}|>1$.

Table S4. RNA-seq data for regulated BGCs in *A. fumigatus*. Sheet 1: Transcriptome data analysis of significantly regulated BGCs in Δ*csdA* mutant. Sheet 2: Transcriptome data analysis of significantly regulated BGCs in Δ*laeB* mutant. Sheet 3: Transcriptome data analysis of BGCs co-regulated by CsdA and LaeB.

Table S5. Information on reported clinical *A. fumigatus* isolates. Used to analyze the amino acid sequence conservation of CsdA and LaeB proteins in the genomes of previously reported clinical isolates.

Table S6. Strains, plasmids, and primers used in this study. Sheet 1: Strains used in this study. Sheet 2: Plasmids used in this study. Sheet 3: Primers used in this study.
